# Network pharmacological prediction on metabolites of dominant endophytic strains from *Salvia plebeia* R. Br.

**DOI:** 10.3389/fmicb.2026.1847318

**Published:** 2026-06-29

**Authors:** Longfei Zhao, Mengjie Liu, Yihan Zhao, Yajun Xu, Jingyi Zhang, Xiaolong Xing, Weiyi Song, Min Li, Jingya Yang

**Affiliations:** 1 Key Laboratory on Agricultural Microorganism Resources Development of Shangqiu, College of Biology and Food, Shangqiu Normal University, Shangqiu, China; 2 College of Life Sciences, Henan Normal University, Xinxiang, China; 3 College of Life Science, Shihezi University, Shihezi, China; 4 College of Life Sciences, Northeast Forestry University, Harbin, China

**Keywords:** biological control, endophyte, metabolomics, network pharmacology, *Salvia plebeia* R. Br.

## Abstract

*Salvia plebeia* R. Br. exerts favorable therapeutic effects on multiple diseases such as bronchitis, urticaria and colorectal cancer. Endophytes play vital roles in the growth and development of medicinal plants. They can not only promote plant growth, but also synthesize active ingredients identical to those of host plants, as well as induce hosts to produce active components. For the first time, this study systematically investigated the endophytes of *S. plebeia*. We evaluated the antagonistic activity and plant growth-promoting traits of endophytes, explored the bacteriostatic effects of strain fermentation broths and volatile organic compounds, and screened strains with excellent functional properties. Headspace solid-phase microextraction coupled with gas chromatography–mass spectrometry (HS-SPME-GC–MS) was adopted to analyze the differences in metabolic components between *S. plebeia* and its dominant endophytes. Combined with network pharmacology, we further elucidated the potential molecular mechanisms by which their active ingredients intervene in diseases. Network pharmacology analysis revealed that *S. plebeia* and its dominant endophytes share a variety of active metabolites, which exert synergistic regulatory effects through common core targets and key signaling pathways. Meanwhile, endophytes possess unique regulatory pathways, a more complex protein–protein interaction network and stronger target binding capacity. They perform medicinal functions via a synergistic mode of multiple components, multiple targets and multiple pathways. This is the first study to apply network pharmacology to predict the correlation of metabolic components between endophytes and their host plants.

## 1 Introduction

Medicinal plants are widely valued for their bioactive components and therapeutic effects, but their quality and yield are significantly affected by both abiotic factors (e.g., temperature, light, humidity, and soil conditions) and biotic factors, among which endophytes have attracted increasing attention (Namdar et al., 2019). First proposed by de Bary (1866), endophytes refer to a group of microorganisms that colonize the internal tissues or cells of healthy plants without causing obvious pathogenic symptoms, including bacteria, fungi, and actinomycetes (Abo-Koura, 2023). Through long-term co-evolution, endophytes establish a mutualistic relationship with their hosts, which can promote plant growth, enhance stress resistance, and produce a variety of bioactive compounds with antibacterial, antifungal, and anticancer properties, showing great potential in agriculture, pharmacy, and biotechnology (Sebola et al., 2020).

*Salvia plebeia* R. Br., a biennial herb of the genus *Salvia* in the Lamiaceae family, is a traditional commonly used Chinese medicinal herb. According to records in traditional Chinese medicine classics, it exerts the effects of clearing away heat and detoxifying, cooling blood and promoting diuresis, and is widely used for treating diseases related to heat toxin in the respiratory tract, which is consistent with the clinical intervention strategies for bronchitis caused by airway inflammation and accumulated heat toxin. Modern clinical and pharmacological studies have demonstrated that extracts of *S. plebeia* can alleviate airway inflammatory injury, inhibit the release of inflammatory factors and relieve respiratory discomfort, and possess certain intervention effects on acute and chronic bronchitis (Liang et al., 2020). In addition, existing studies have found that active ingredients such as flavonoids and phenolic acids in *S. plebeia* can ameliorate insulin resistance, regulate glucose and lipid metabolism, and mitigate oxidative and inflammatory damage to the body to a certain extent, showing potential intervention effects on type 2 diabetes (Wang et al., 2026).

It has been confirmed that endophytes of medicinal plants co-evolve with their hosts and produce bioactive metabolites with antioxidant and anti-inflammatory activities. Such metabolites can help enhance the stress and disease resistance of host plants and thus have potential agricultural application values. Meanwhile, they are expected to regulate disorders such as inflammatory disturbance and metabolic abnormality in the human body (Gao et al., 2025). Relevant studies have verified that endophytes of medicinal plants are capable of synthesizing bioactive metabolites with novel chemical structures. A typical example is the taxol-producing endophytes isolated from *Taxus brevifolia* (Shan et al., 2022).

At present, the metabolic interaction mechanisms between medicinal plants and their endophytes have not been systematically and clearly elucidated, which may be attributed to the large variety of metabolic components, complex symbiotic interaction processes and intricate action networks. The relevant research system still needs to be further improved. On this basis, after preliminarily clarifying the agricultural biocontrol and plant growth-promoting functions of endophytes from *S. plebeia* and screening out dominant functional strains, this study adopted network pharmacology to preliminarily predict the potential molecular mechanisms of these endophytes acting on typical inflammatory and metabolic diseases, aiming to provide a fundamental reference for exploring the metabolic component interaction mechanisms of endophytes from *S. plebeia*.

## 2 Materials and methods

### 2.1 Area of study and sampling

Healthy and disease-free *S. plebeia* R. Br. samples were collected from nine sites in Henan, Jiangsu and Shandong Provinces from July to August 2024 and used as experimental materials. Detailed sample information is presented in Supplementary Table S1. All plant specimens were identified by Dr. Min Li at Key Laboratory on Agricultural Microorganism Resources Development of Shangqiu, and numbered voucher specimens are preserved in this laboratory.

### 2.2 Isolation of endophyte, media and growth conditions

Fresh *S. plebeia* with soil was collected using sterile shovels, placed in sterile self-sealing bags, transported in a vehicle-mounted freezer, and processed for endophyte isolation within 24 h. After thorough rinsing with sterile water to remove surface impurities, 1 g roots, stems, and leaves were separately excised under sterile conditions. Surface sterilization was performed as follows: immersion in 75% ethanol for 5 min (rinsed 3–5 times with sterile water), then 4% available chlorine sodium hypochlorite for 2 min (rinsed 8–10 times) (Anjum and Chandra, 2015). The final rinse solution was spread on beef extract peptone (BEP) medium as a sterility control. Sterilized tissues were ground into homogenate and serially diluted to 10^−2^, 10^−3^, 10^−4^, and 100 μL of each dilution was spread on BEP solid medium, followed by incubation at 37 °C for 48 h. Single colonies with distinct morphologies were selected, streaked, and re-incubated. Pure cultures were confirmed by Gram staining and microscopic observation (consistent bacterial size and staining) (Mehmood et al., 2021), then propagated on BEP slants. Purified strains were cultured in LB liquid medium (37 °C, shaking), adjusted to OD_600_ = 1 with sterile water for subsequent experiments, and preserved in 40% glycerol tubes at −80 °C (1 mL aliquots of LB cultures).

Strict strain culture and preservation protocols were applied to maintain endophyte stability and prevent functional decline. All strains were subcultured no more than three times, preserved via glycerol cryopreservation and slant refrigeration, and freshly activated before tests. Uniform culture conditions ensured stable antibacterial and growth-promoting activities.

### 2.3 Functional traits

To evaluate the physiological, biochemical properties and functional potential of the isolated strains, the following tests were conducted in triplicate for each strain unless otherwise specified. The experimental procedures are described as follows: (1) Protease activity assay (Anjum and Chandra, 2015). (2) Amylase activity assay. (3) Cellulase activity assay (Al Talebi et al., 2022). (4) Chitinase activity assay (Kuddus and Ahmad, 2013). (5) Catalase test. (6) Phosphate-solubilizing capacity assay (Matos et al., 2017). (7) Nitrogen-fixing capacity assay (Zhang et al., 2022). (8) Siderophore secretion assay (Li et al., 2023). (9) IAA (Indole-3-Acetic Acid) production assay (Phetcharat and Duangpaeng, 2012). (10) ACC deaminase activity assay (Passari et al., 2016; Cui et al., 2019). (11) Biofilm formation assay (Kamimura et al., 2022).

### 2.4 Antagonism assay against pathogenic fungi

(1) Antifungal activities of endophytes against *Magnaporthe oryzae*, *Fusarium oxysporum*, and *Fusarium graminearum* (provided by Key Laboratory on Agricultural Microorganism Resources Development of Shangqiu) were determined by the cross confrontation plate method (FiraFiras et al., 2024). Pathogenic fungi were inoculated on PDA medium, cultured at 28 °C for 5 days, and 5 mm mycelial discs were punched and inoculated at the center of fresh PDA plates (3 replicates). After 7 days, indicator mycelial discs were re-punched to new PDA plates’ center; 2.5 μL endophyte suspension (OD_600_ = 1) was spotted at 4 points 2.5 cm from the center, with non-inoculated group as blank control. All plates were inverted and cultured at 28 °C in dark for 7 days to observe inhibition zones. For primary screening, multiple samples were tested per Petri dish; strains with obvious activity were re-screened (single strain per dish), sealed with parafilm, and incubated under the same conditions for 7 days. Radius of pathogenic fungi (*R*) was measured, and inhibition rate was calculated according to the following formula:

$$\text{Inhibition Rate}(\%)=\frac{R_{\text{control}}-R_{\text{treatment}}}{R_{\text{control}}}\times 100\%$$

(2) The antifungal activity of cell-free fermentation broth from *S. plebeia* endophytes against pathogenic fungi was determined by the method of Li et al. (2025) with minor modifications. Briefly, 40 mL sterile LB liquid medium (in 250 mL Erlenmeyer flasks) was inoculated with 1% endophytic bacterial suspension (OD_600_ = 1), followed by fermentation at 37 °C and 180 r/min for 48 h. The fermentation broth was centrifuged at 12,000 r/min and 4 °C for 10 min; the supernatant was filtered through a 0.22 μm sterile filter membrane. Then 10 mL filtrate was added to PDA solid medium cooled to 80 °C, with equal volume of sterile LB medium as control. After solidification, a 5 mm-diameter mycelial disc was inoculated at each plate center (3 replicates per strain). Plates were sealed with parafilm, inverted, and cultured in dark at 28 °C for 7 days. The growth diameter of pathogenic fungi (*D*) was measured, and inhibition rate was calculated using the formula below:

$$\text{Inhibition Rate}(\%)=\frac{D_{\text{control}}-D_{\text{treatment}}}{D_{\text{control}}}\times 100\%$$

(3) The antifungal activity of volatile organic compounds (VOCs) produced by *S. plebeia* endophytes was determined using the plate-to-plate confrontation method described by Gao et al. (2017). Briefly, 100 μL of endophyte suspension (OD_600_ = 1) was spread evenly on beef extract peptone agar. A 5-mm mycelial disc of each pathogenic fungus was placed at the center of a PDA plate. The two agar surfaces were sealed face-to-face with the PDA plate on top, and incubated at 28 °C in darkness for 7 days. Non-inoculated plates served as blank controls, with three replicates per treatment. VOC-mediated antifungal effects were assessed by comparing colony growth with the control group. Hyphal samples were collected from the colony margin for morphological observation under an inverted microscope, and the inhibition rate was calculated using the aforementioned formula.

### 2.5 DNA extraction, PCR-based fingerprinting, and molecular identification

Total DNA of dominant strains was extracted. The 50 μL PCR system contained 1 μL template DNA, 25 μL 2 × Taq mix (Sangon Biotech), 1 μL each of bacterial universal primers P1 and P6, and 22 μL dd H_2_O (Shreshtha et al., 2024). The 16S rRNA amplification program was as follows: initial denaturation at 94 °C for 3–5 min; 30 cycles of 94 °C for 45 s, 55 °C for 1 min and 72 °C for 90 s; final extension at 72 °C for 20 min, followed by storage at 4 °C. PCR products were sequenced by General Biosystems (Anhui). Obtained sequences were blasted in GenBank, and homologous 16S rDNA sequences were retrieved. After trimming invalid terminals via MEGA 11.0, the phylogenetic tree was constructed using the NJ method, and node stability was assessed with 1,000 bootstrap replicates (Cheng et al., 2022).

### 2.6 Determination of bioactive compounds from *Salvia plebeia* and its endophytes

#### 2.6.1 Determination of antibacterial and antioxidant capacities of *Salvia plebeia* against pathogenic bacteria

The underground and aboveground parts of *S. plebeia* were air-dried, ground separately, and sieved through a 40-mesh sieve. Dry powder samples (weighing 20, 25, and 30 g, respectively) were extracted with 400 mL of 80 °C hot water under ultrasonication (40 kHz) in an 80 °C water bath for 1 h. After standing and centrifugation (8,000 r/min, 10 min), supernatants were collected, and the extraction was repeated twice more. Combined supernatants were filtered, concentrated to 5 mL via rotary evaporation (final concentrations: 4, 5, and 6 g/mL), and sterilized through a 0.22 μm membrane to obtain sterile aqueous extracts. *Escherichia coli*, *Bacillus subtilis*, and *Staphylococcus aureus* were cultured to the logarithmic phase at 37 °C with shaking (180 r/min), and their OD_600_ was adjusted to 1. Bacterial suspensions (100 μL) were spread on beef extract peptone agar plates. Sterile 5 mm filter paper discs soaked in extracts were placed on inoculated plates, with sterile water as the blank control. All plates were incubated at 37 °C for 24 h, and the diameters of inhibition zones were measured and recorded.

Accurately weighed 20 g root and leaf samples of *S. plebeia* were separately ultrasonically extracted with 400 mL hot water at 80 °C for 60 min. The extracts were filtered and centrifuged at 6000 r/min, and the residues were re-extracted twice under the same conditions. All combined extracts were freeze-dried into powder, which was prepared into sample solutions with concentrations of 0.2, 0.4, 0.6, 0.8, 1.0, 1.5 and 2.0 mg/mL. The *in vitro* antioxidant activity of samples was determined by DPPH and ABTS^+^ radical scavenging assays (Giri et al., 2023). The DPPH and ABTS^+^ free radical scavenging rates (*I*%) were calculated according to the following formula:


$$I(\%)=(1-\frac{A_{0}}{A_{S}})\times 100\%$$

Where *A_S_* = absorbance of the sample tube; *A*_0_ = absorbance of the control tube.

#### 2.6.2 Determination of antibacterial and antioxidant capacities of endophytes from *Salvia plebeia* against pathogenic bacteria

The paper disc diffusion method (Hidayat et al., 2023) was utilized to evaluate the antagonistic activity of endophytic strains isolated from *S. plebeia* against *S. aureus*, *E. coli*, and *B. subtilis*. The DPPH and ABTS^+^ free radical scavenging rates (*I*%) were determined using the same assay methods and calculation formulas as described above.

#### 2.6.3 Identification of active components in *Salvia plebeia* and its endophytes

Fresh *S. plebeia* samples were collected from 9 regions, with more than 3 healthy and uniformly grown individuals mixed to form one regional sample. After rinsing with tap water and deionized water, samples were divided into aboveground and underground parts. A 2.00 g aliquot of each part was sliced, sealed in a 20 mL headspace vial, and subjected to volatile organic compound (VOC) analysis using headspace solid-phase microextraction coupled with gas chromatography–mass spectrometry (HS-SPME-GC–MS) (Jayakumar et al., 2021). A 50 μm PDMS fiber was used with a constant insertion depth of 1.5 cm; samples were pre-equilibrated at 80 °C for 20 min and extracted at the same temperature for 30 min before desorption. Chromatographic separation was performed on an HP-5 MS capillary column (30 m × 0.25 mm × 0.25 μm) with splitless injection at 230 °C and high-purity helium as carrier gas (1.0 mL/min). The oven temperature program was carried out as follows: 40 °C held for 2 min, increased to 62 °C at 2 °C/min and held for 2 min, raised to 140 °C at 20 °C/min and held for 2 min, then finally elevated to 230 °C at 20 °C/min and held for 2 min. Mass spectrometry was operated in electron ionization (EI) mode at 70 eV, with a scanning range of 30–400 m/z, ion source at 230 °C, quadrupole at 150 °C, and peak identification using the NIST 20. L database. VOCs produced by 6 superior endophytic strains isolated from *S. plebeia* were also analyzed according to the identical HS-SPME-GC–MS procedure. Beef extract peptone agar was autoclaved at 121 °C for 20 min, and 3 mL was transferred into 20 mL headspace vials tilted at 15° to form slants. After solidification, 100 μL of bacterial suspension (OD_600_ = 1) was spread onto each slant, sealed immediately, and incubated at 37 °C in the dark for 2 days. Uninoculated medium served as blank control, and all treatments included three biological replicates (López et al., 2021). Prior to extraction, vials were equilibrated at room temperature for 20 min. For chemical composition analysis of *S. plebeia* extracts, healthy and pest-free plants were rinsed, separated into aboveground and underground parts, shade-dried for 7 days, and further dried to constant weight at 45 °C. Dried materials were ground, passed through a 40-mesh sieve, and 10 g powder was soaked in 50 mL ethyl acetate (solid–liquid ratio 1:5) for 12 h, followed by ultrasonic-assisted extraction at 300 W, 40 kHz, 25 °C for 2 h with shaking every 30 min. The mixture was filtered, and the filtrate was concentrated to near dryness using a rotary evaporator (60 °C, 0.08 MPa, 60 rpm). One milliliter of concentrate was filtered through a 0.22 μm organic membrane and transferred to GC–MS vials, with ethyl acetate alone as blank control (Jabali et al., 2019). GC was performed with high-purity helium carrier gas (1.0 mL/min), injection port at 250 °C, split ratio 10:1, injection volume 1 μL, and temperature program was carried out as follows: 40 °C held for 1 min, increased to 240 °C at 3 °C/min and held for 5 min. MS parameters included transfer line at 250 °C, ion source at 230 °C, 70 eV EI, scanning range 35–500 m/z, and peak identification using the NIST20. L database. For secondary metabolite analysis of the 6 endophytic strains, strains were activated in LB liquid medium; 3 mL of activated culture was inoculated into 300 mL LB medium and incubated at 37 °C and 180 r/min for 24 h. After centrifugation at 8000 r/min for 10 min, supernatants were concentrated under vacuum at 60 °C (90 rpm) and freeze-dried. The powder was dissolved in ethyl acetate, centrifuged at 12,000 r/min for 10 min, and 1 mL of supernatant was filtered through a 0.22 μm membrane for GC–MS analysis. Uninoculated LB medium subjected to the same extraction procedure was adopted as blank control (Tapfuma et al., 2020), and the GC–MS conditions were identical to those used for *S. plebeia* extracts.

### 2.7 Network pharmacology analysis of active ingredients

**Figure 1:**
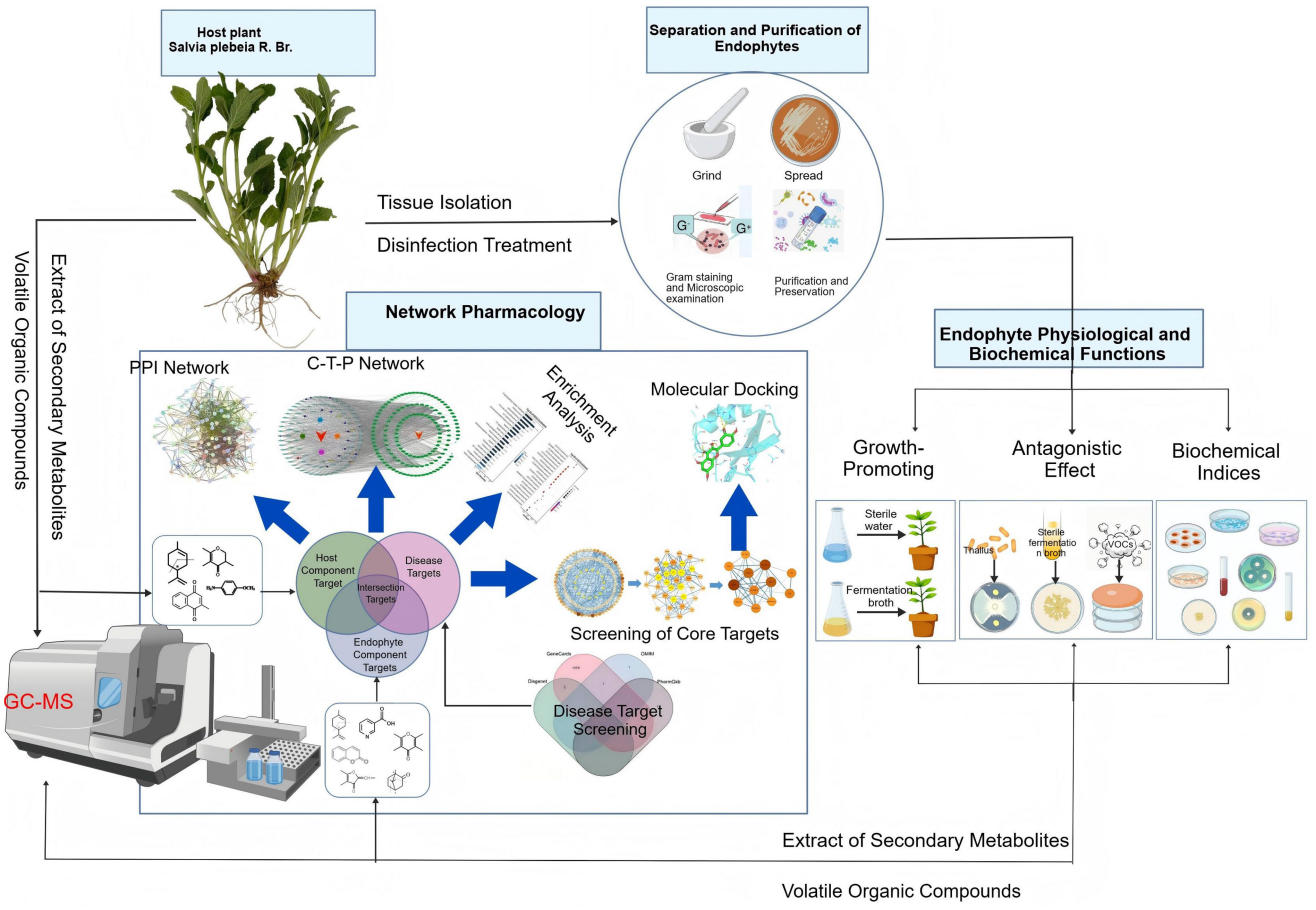
Metabolomics-based network pharmacology analysis of *Salvia plebeia*-endophyte interactions and the physiological and biochemical functions of the endophytes.

In this study, multi-database joint analysis combined with bioinformatics techniques was adopted. Firstly, HS-SPME-GC–MS was used to identify the chemical constituents of *S. plebeia* and its endophytes, and components with mass spectral matching degree ≥70% were screened. The standardized SMILES structures of each component were obtained from the PubChem database^1^ and imported into Swiss Target^2^ Prediction to predict potential targets of chemical constituents. Subsequently, Type 2 diabetes mellitus and bronchitis were used as keywords to retrieve disease-related targets from GeneCards,^3^ OMIM^4^ and TTD^5^ databases. The overlapping core targets were acquired by intersecting compound targets with disease targets. The STRING^6^ database was used for protein–protein interaction (PPI) analysis with human species and a high confidence score of 0.9. Core targets were screened according to degree centrality and betweenness centrality, and the PPI visual network was constructed via Cytoscape 3.9.1 software. The key intersecting targets were uploaded to the DAVID database.^7^ With *p* < 0.05 as the screening threshold, GO functional annotation and KEGG pathway enrichment analysis were performed to clarify biological functions and core enriched pathways. Finally, the compound-target-disease and target–pathway visual networks were established by Cytoscape. Topological parameters were calculated using the CytoNCA plugin, so as to elucidate the synergistic mechanism of this plant and its endophytes against the two diseases through multi-component, multi-target and multi-pathway regulation. Common core target proteins were selected as receptors from the “component-target–pathway” (C–T–P) network and protein–protein interaction (PPI) network. Meanwhile, the active components with the highest correlation with the core target proteins were selected as ligands. The 2D structure files of small-molecule ligands (SDF format) and protein receptors (PDB format) were downloaded from PubChem and Uniprot,^8^ respectively. The CB-Dock2 web server^9^ was employed to perform molecular docking experiments to simulate the interactions between ligands and receptors. After docking, PyMOL 3.1.8 software was applied to analyze the molecular docking results. The binding status between ligands and receptors was judged based on the binding energy values. A binding energy ≤−5.0 kcal/mol indicates favorable binding between the ligand and the receptor, whereas a binding energy ≤−7.0 kcal/mol indicates strong binding activity (Su et al., 2024). Molecular docking verification further validated the interactions between the active components of the metabolites of *S. plebeia* and its endophytes and the key targets (see Figure 1).

### 2.8 Statistical analysis

Data were analyzed using SPSS 26.0. Two-group differences were assessed via *t*-test, and multi-group differences via one-way ANOVA with Duncan’s test. All assays were repeated three times, and results were expressed as mean ± SEM. Different *p*-value thresholds were set for different experiments.

## 3 Results

### 3.1 Isolation statistics of endophytes in *Salvia plebeia* from multiple regions

A total of 204 endophytic bacteria were isolated from the roots, stems and leaves of *S. plebeia*, including 118 from roots, 36 from stems and 50 from leaves. 4 strains (R111, R112, S32, S34) with growth-promoting potential were screened based on physicochemical characteristics of *S. plebeia* secretions. Detailed data are shown in Supplementary Figure 1.

### 3.2 Antagonism of endophyte

#### 3.2.1 Determination of antifungal activity of endophytes from *Salvia plebeia* against phytopathogenic fungi

**Figure 2:**
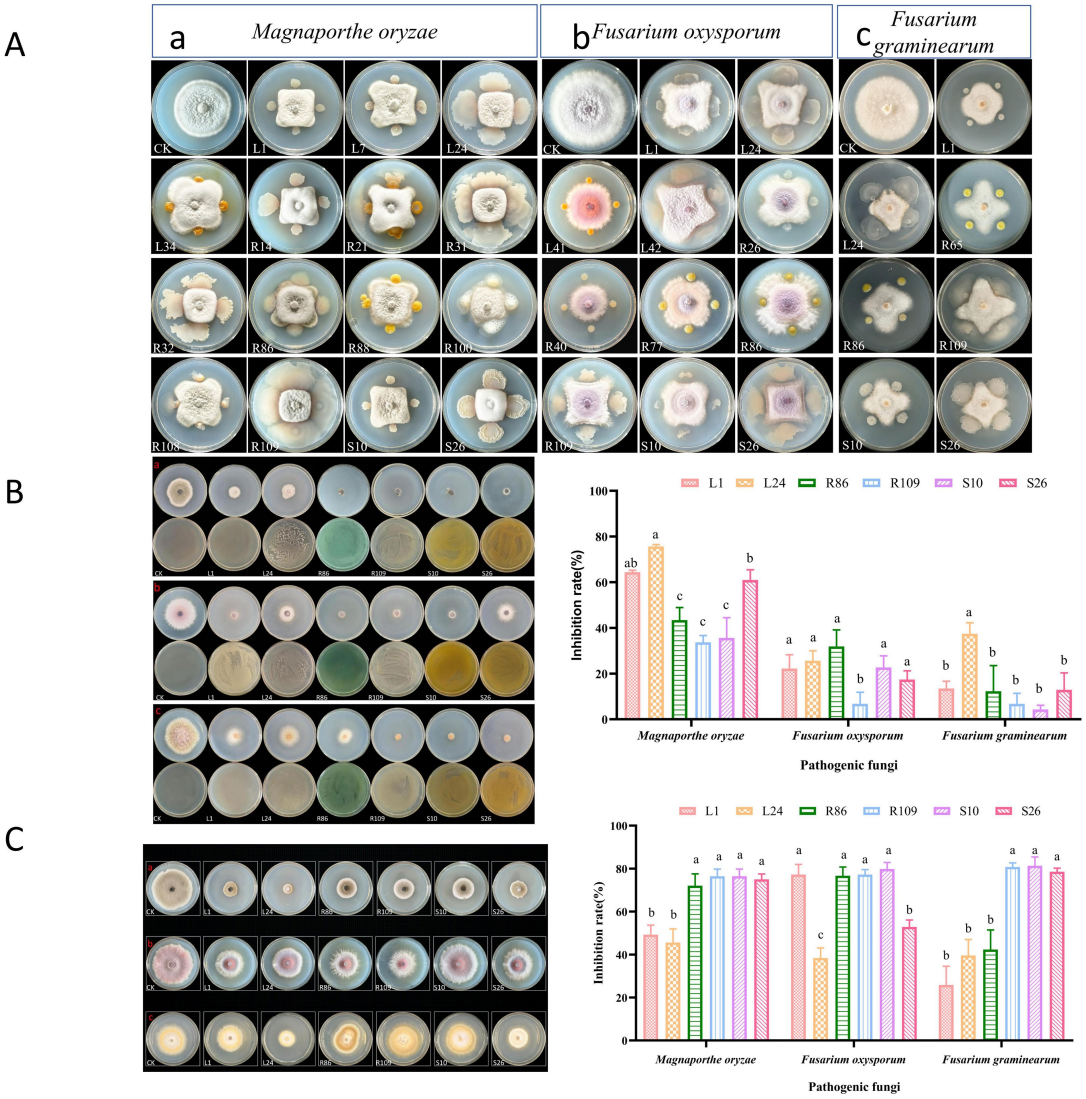
Antifungal activities of endophytic bacteria and their metabolites from *Salvia plebeia* against three pathogenic fungi. **(A)** Endophytic strains with antagonism rate ≥ 50%: **a**, *Magnaporthe oryzae*; **b**, *Fusarium oxysporum*; **c**, *Fusarium graminearum*. **(B)** Antifungal effects of fermentation broth and inhibition rates of sterile fermentation filtrate of antagonistic endophytic bacteria: **a**, *Magnaporthe oryzae*; **b**, *Fusarium oxysporum*; **c**, *Fusarium graminearum*. **(C)** Antifungal effects of volatile organic compounds (VOCs) produced by antagonistic endophytic bacteria: **a**, *Magnaporthe oryzae*; **b**, *Fusarium oxysporum*; **c**, *Fusarium graminearum*. Different lowercase letters indicate significant differences among treatments at *p* < 0.05.

Among 204 endophytic bacterial strains from *S. plebeia*, 28 strains (13.73%) showed antagonistic activity against *M. oryzae*, 15 of which had an inhibition rate exceeding 50% (L1, L7, L24, L34, R14, R21, R31, R32, R86, R88, R100, R108, R109, S10, S26); strain R109 had the most significant inhibitory effect with an inhibition rate of 61% [Figures 2A(a)]. A total of 47 strains (23.04%) displayed antagonistic activity against *F*. *oxysporum*, 11 of which had an inhibition rate over 50% (L1, L24, L41, L42, R26, R40, R77, R86, R109, S10, S26); strain L24 had the most prominent inhibitory effect with an inhibition rate of 59% [Figures 2A(b)]. For *F*. *graminearum*, 42 antagonistic strains were identified (20.59%), 7 of which had an inhibition rate higher than 50% (L1, L24, R65, R86, R109, S10, S26); strain S26 presented the most remarkable inhibitory effect with an inhibition rate of 61% [Figures 2A(c)]. 6 endophytic bacterial strains (L1, L24, R86, R109, S10 and S26) all exhibited an inhibition rate of more than 50% against the three tested phytopathogenic fungi. Consequently, these 6 antagonistic endophytic bacterial strains were selected for subsequent experiments.

#### 3.2.2 Determination of antifungal activity of sterile fermentation broths from *Salvia plebeia* endophytes against phytopathogenic fungi

6 antagonistic strains (L1, L24, R86, R109, S10, and S26) all inhibited the mycelial growth of three plant pathogenic fungi. Figure 2B shows the colony morphology of *M. oryzae* [Figures 2B(a)], *F*. *oxysporum* [Figures 2B(b)], and *F*. *graminearum* [Figures 2B(c)] after 7 days of culture treated with sterile fermentation broths. Compared with the control group, the treatment groups supplemented with fermentation broths of antagonistic strains showed slow and sparse mycelial growth. The experimental results showed that the inhibition rates of strains L1, L24, R86, R109, S10, and S26 against *M. oryzae* were 64, 76, 43, 34, 361, and 61%, respectively. Among them, L1 and L24 exhibited significantly higher antifungal activity than the other endophytic strains (*p* < 0.05), with no significant difference between L1 and L24 (*p* ≥ 0.05). For *F. oxysporum*, the inhibition rates of the 6 strains were 22, 26, 32, 7, 23, and 17%, respectively. Strain R86 showed the highest antifungal activity, which was not significantly different from L1, L24, S10, and S26 (*p* ≥ 0.05) but significantly higher than R109 (*p* < 0.05). Against *F. graminearum*, the inhibition rates were 14, 37, 12.27, 75, 4, and 13%, respectively, with L24 showing significantly higher antifungal activity than the other endophytic bacteria (*p* < 0.05).

#### 3.2.3 Determination of antifungal activity of volatile organic compounds (VOCs) from endophytes of *Salvia plebeia* against phytopathogenic fungi

The double-dish sealing method was used to detect the antifungal activity of volatile organic compounds produced by 6 antagonistic strains against *M. oryzae*, *F*. *oxysporum* and *F*. *graminearum*. The results (Figure 2C) showed that the metabolites exerted remarkable inhibitory effects on the three pathogens, proving that the tested strains could synthesize and secrete antifungal volatile substances.

The volatile compounds produced by antagonistic endophytes showed inhibition rates of 9, 46, 72, 75, 76 and 76% against *M. oryzae*. Strains R86, R109, S10 and S26 exhibited significantly stronger antifungal activity than L1 and L24 (*p* < 0.05). Against *F*. *oxysporum*, the inhibition rates were 77, 38, 77, 77, 80 and 53%. Strains L1, R86, R109 and S10 presented superior antifungal effects (*p* < 0.05). For *F*. *graminearum*, the corresponding inhibition rates were 26, 30, 42, 89, 81 and 79%, and strains R109, S10 and S26 displayed markedly higher inhibitory activity (*p* < 0.05).

### 3.3 Identification and phylogenetic analysis of isolates and plant growth promotion

#### 3.3.1 Identification of superior strains

**Figure 3:**
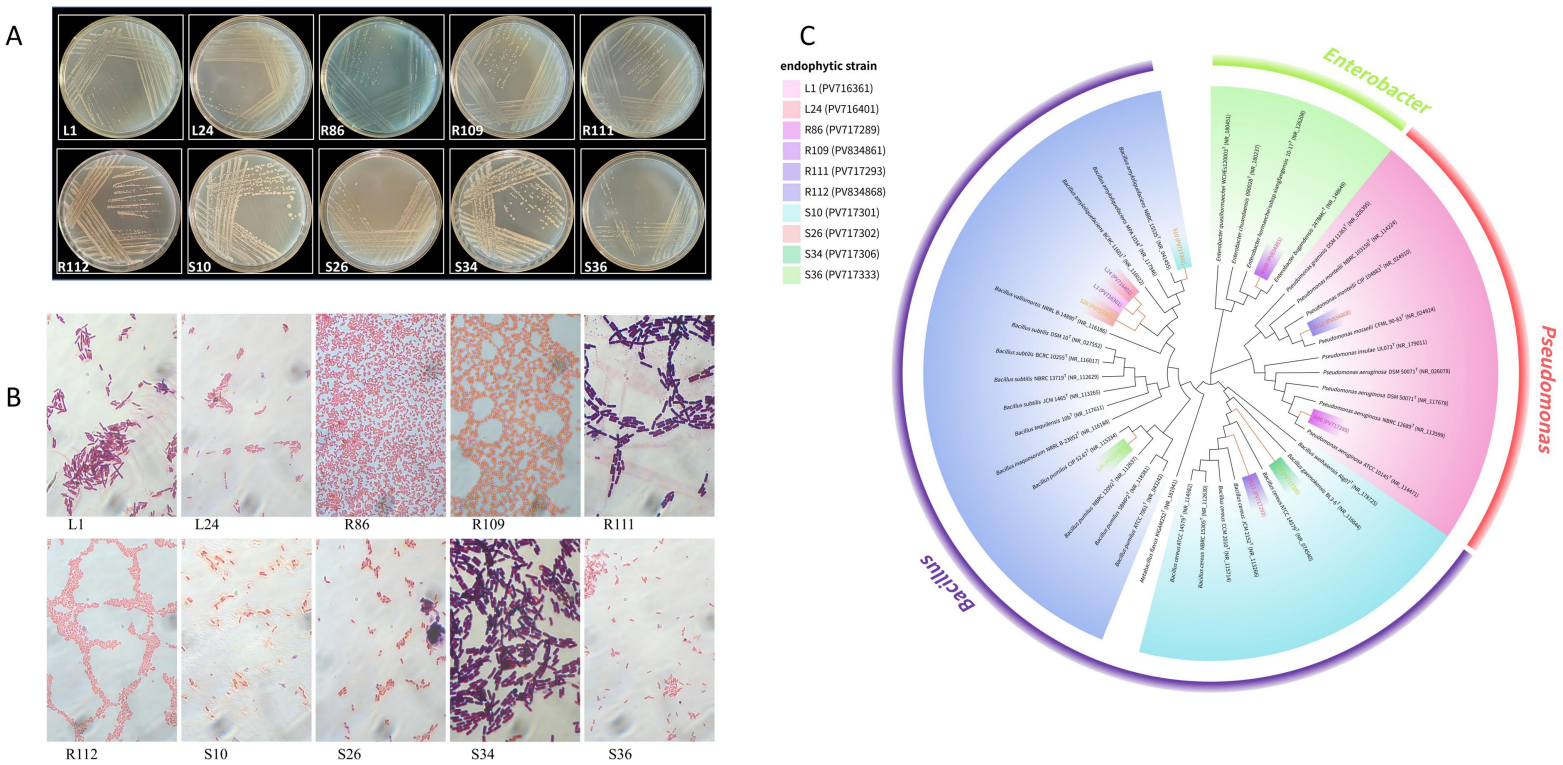
Identification of superior endophytic strains from *Salvia plebeia*. **(A)** Morphological characteristics of dominant strains’ colonies isolated from *Salvia plebeia*. **(B)** Gram staining results of dominant strains isolated from *Salvia plebeia*, scale bar = 1 μm. **(C)** Phylogenetic tree constructed based on 16S rRNA gene sequences of dominant endophytic bacteria from *Salvia plebeia*.

In this study, 10 endophytic bacteria with plant growth-promoting and antifungal activities were isolated from *Salvia plebeia*: L1 (PV716361), L24 (PV716401), R86 (PV717289), R109 (PV834861), R111 (PV717293), R112 (PV834868), S10 (PV717301), S26 (PV717302), S34 (PV717306), and S36 (PV717333). Their colony morphology and Gram staining are shown in Figures 3A,B, respectively. Strains L1, L24, R111, S10, S26, S34, and S36 were Gram-positive; R86, R109, and R112 were Gram-negative. A 16S rRNA phylogenetic tree was constructed (Figure 3C), and three major clades were identified. Based on morphology and phylogenetic analysis: L1, L24, S10, and S26 were identified as *Bacillus amyloliquefaciens*; S36 as *Bacillus pumilus*; R111 and S34 as *Bacillus cereus*; R86 as *Pseudomonas aeruginosa*; R112 as *Pseudomonas mosselii*.

### 3.4 Determination of bioactive compounds from *Salvia plebeia* and its endophytes

#### 3.4.1 Determination of antibacterial and antioxidant capacities of *Salvia plebeia* against pathogenic bacteria

**Figure 4:**
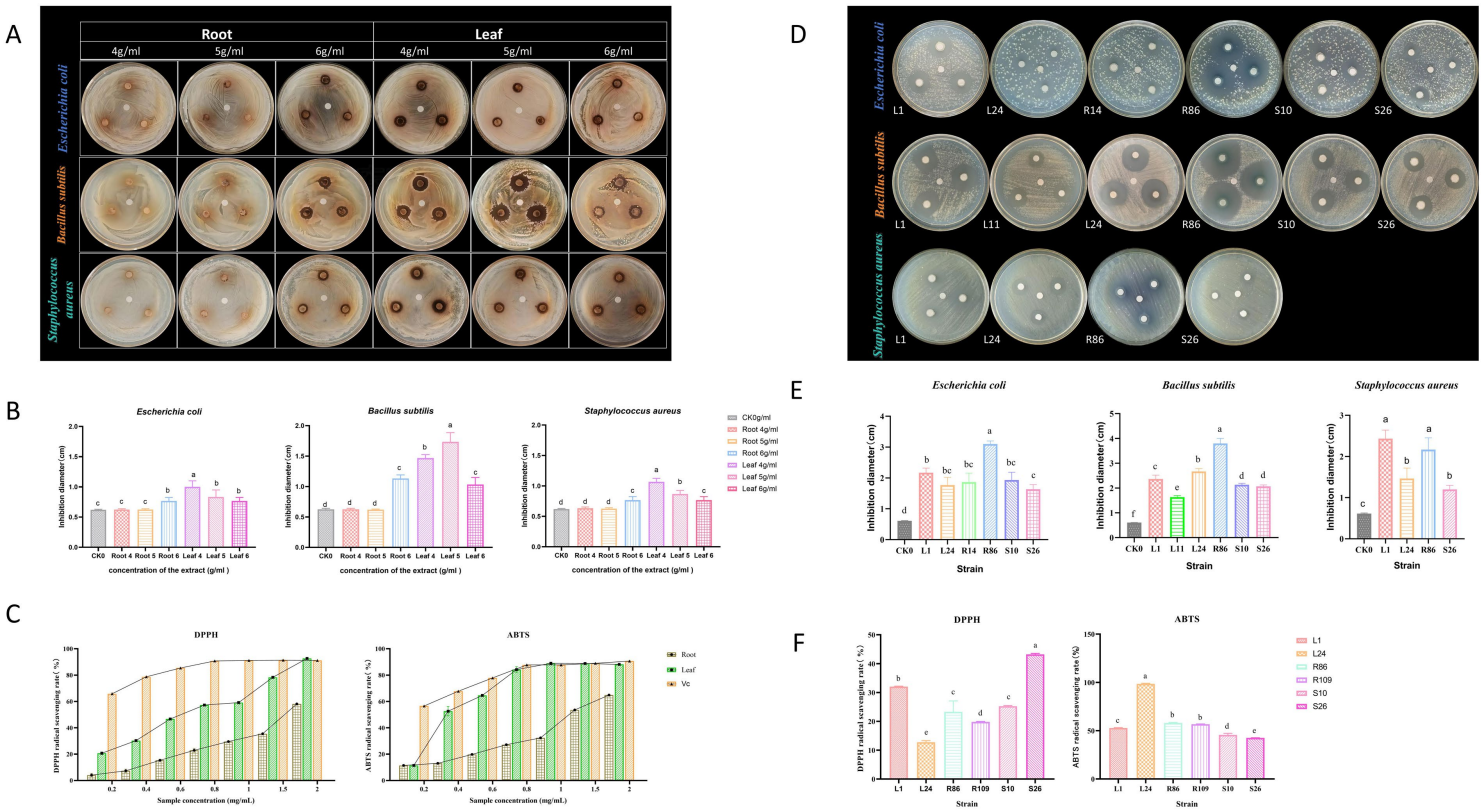
Antibacterial and antioxidant activities of *Salvia plebeia* and its endophytes against pathogenic bacteria. **(A)** Antibacterial effects of *S. plebeia* tissue extracts at different concentrations on various pathogenic bacteria. **(B)** Comparison of antibacterial activities of *S. plebeia* tissue extracts with different concentrations against various pathogenic bacteria. **(C)** DPPH radical and ABTS^+^ radical scavenging rates of extracts from roots and leaves of *S. plebeia*. **(D)** Antibacterial effects of endophytes isolated from *S. plebeia* with antibacterial activity on different pathogenic bacteria. **(E)** Comparison of antibacterial activities of *S. plebeia* endophytes against various pathogenic bacteria. **(F)** DPPH radical and ABTS^+^ radical scavenging rates of six dominant endophytic strains from *S. plebeia*. (Different lowercase letters indicate significant differences among treatment groups at *p* < 0.05).

As shown in Figure 4A, the root and leaf extracts of *S. plebeia* (at concentrations of 4, 5, and 6 g/mL) exhibited antibacterial activity against *Escherichia coli*, *Bacillus subtilis*, and *Staphylococcus aureus*. Both extracts inhibited the three pathogenic bacteria to varying degrees, and the leaf extract showed stronger antibacterial activity than the root extract at the same concentration. At the statistical level of *p* < 0.05 (Figure 4B), the root extracts at 4 and 5 g/mL showed no significant difference from the control group against the three pathogens, indicating no obvious antibacterial effect at these concentrations. As shown in Figure 4C, within the concentration range of 0.2–2 mg/mL, the DPPH and ABTS^+^ radical scavenging capacities of the root and leaf extracts of *S. plebeia* significantly increased with rising concentration, showing an obvious concentration-dependent manner.

#### 3.4.2 Determination of antibacterial and antioxidant capacities of endophytes from *Salvia plebeia* against pathogenic bacteria

As shown in Figure 4D, the isolated and purified endophytes from *S. plebeia* exhibited antibacterial activity against *Escherichia coli*, *Bacillus subtilis*, and *Staphylococcus aureus*. The differential analysis in Figure 4E showed that, at the *p* < 0.05 level: The antibacterial activity against *E. coli* increased in the order: S26 < L24 < R14 < S10 < L1 < R86; Against *B. subtilis*: L11 < S26 < S10 < L1 < L24 < R86; Against *S. aureus*: L24 < S26 < R86 < L1. 6 superior endophytic strains (L1, L24, R86, R109, S10, S26) were selected for antioxidant activity assay. As shown in Figure 4F, the DPPH radical scavenging rates of the 6 strains ranked from high to low were: S26 > L1 > S10 > R86 > R109 > L24. The ABTS^+^ radical scavenging rates ranked from high to low were: L24 > R86 > R109 > L1 > S10 > S26.

#### 3.4.3 Identification of active components in *Salvia plebeia* and its endophytes

HS-SPME-GC–MS was used to analyze VOCs in roots and leaves of *S. plebeia* (Supplementary Table S2). A total of 65 VOCs were identified (31 in roots, 40 in leaves), with only 6 shared, showing distinct composition for rapid identification. Aromatic compounds were dominant; alcohols (63.55%) were most abundant in roots and peptides (84.05%) in leaves, with multiple bioactive components.

VOCs from 6 antibacterial endophytic strains were analyzed by HS-SPME-GC–MS (Supplementary Table S3). Seventy-nine compounds were detected, only 4 shared among strains. Esters were predominant, with 2-phenylethanol as a major constituent, including tirapazamine and hydroxyurea.

Ethyl acetate extracts of *S. plebeia* roots and leaves were analyzed by liquid injection GC–MS (Supplementary Table S4). Eighty compounds were identified (43 in roots, 51 in leaves), 13 shared, with clear differences. Aromatic compounds were most abundant, esters dominant, containing minoxidil and tirapazamine.

Extracts of endophytic fermentation broths were analyzed by GC–MS (Supplementary Table S5). One hundred seventy-nine compounds were identified, few shared among strains. Aromatic compounds were dominant, with cyclo(Pro-Gly) as a major component, including pirenzepine and trigonelline.

### 3.5 Network pharmacological analysis of *Salvia plebeia* and its endophytic bacteria

#### 3.5.1 Prediction and screening of targets for type 2 diabetes mellitus (T2DM) and bronchitis (BR)

**Figure 5:**
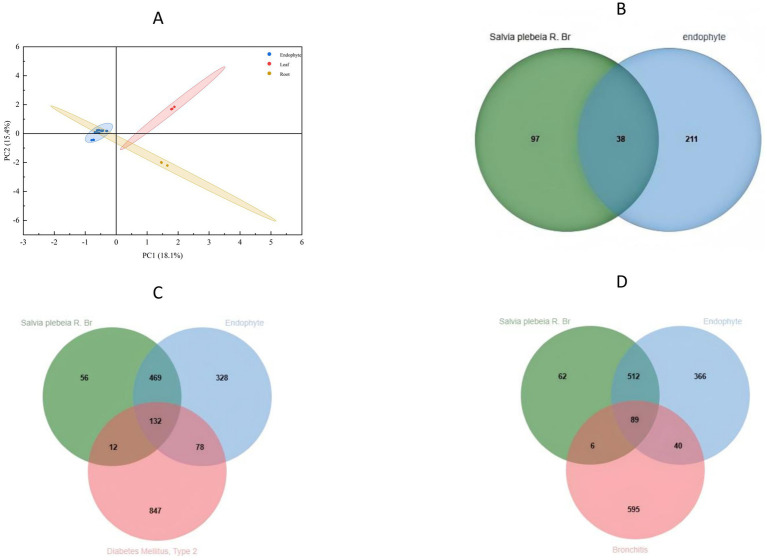
**(A)** Principal component analysis (PCA) of chemical components in roots, leaves and endophytic bacteria of *Salvia plebeia*. **(B)** Venn diagram showing the intersection of active components between *Salvia plebeia* and its endophytic bacteria. **(C)** Venn diagram of the intersection of effective component targets of *Salvia plebeia* and its endophytic bacteria with T2DM disease targets. **(D)** Venn diagram of the intersection of effective component targets of *Salvia plebeia* and its endophytic bacteria with BR disease targets.

Thirty-eight compounds were common between host and endophytes, including hydroxyurea, betaine, and tirapazamine. Figure 5A (PCA) showed clear separation of roots and leaves along PC1, distinct clustering of endophytes and host, with a cumulative contribution of 33.5% (PC1 + PC2).

These results demonstrate significant metabolic differences among roots, leaves, and endophytes of *S. plebeia*, with high discrimination between groups. After removing low-absorption and unidentified components, 40 active substances were detected in volatile components of *S. plebeia*, and 42 were found in ethyl acetate extract. Two common substances were identified, namely acetylated tyramine and 8-hydroxyquinoline N-oxide, with a total of 80 active components in the plant. 44 and 89 active substances were separately identified in volatile components and ethyl acetate extract of endophytic bacteria. Ethyl phenylacetate and acridone acetic acid were the two shared components, and 132 active substances were obtained in total. Eleven identical active components existed between the plant and endophytic bacteria, including n-valeric acid, betaine, 8-hydroxyquinoline N-oxide and 1,4-cyclohexanedione (Figure 5B).

Disease-related targets (after integration and deduplication): For T2DM, 856, 184 and 97 targets were retrieved from GeneCards, TTD and OMIM, with a total of 1,069 unique targets. For BR, 724, 3 and 7 targets were acquired from the above databases, resulting in 730 unique targets.

Intersection results: Intersection between *S. plebeia* targets and T2DM targets: 144. Intersection between endophytic bacteria targets and T2DM targets: 210 (Figure 5C). Intersection between *S. plebeia* targets and BR targets: 95. Intersection between endophytic bacteria targets and BR targets: 129 (Figure 5D). Common targets between total active ingredient targets and T2DM: 132Common targets between total active ingredient targets and BR: 89.

#### 3.5.2 Construction of protein–protein interaction (PPI) network and screening of core targets

**Figure 6:**
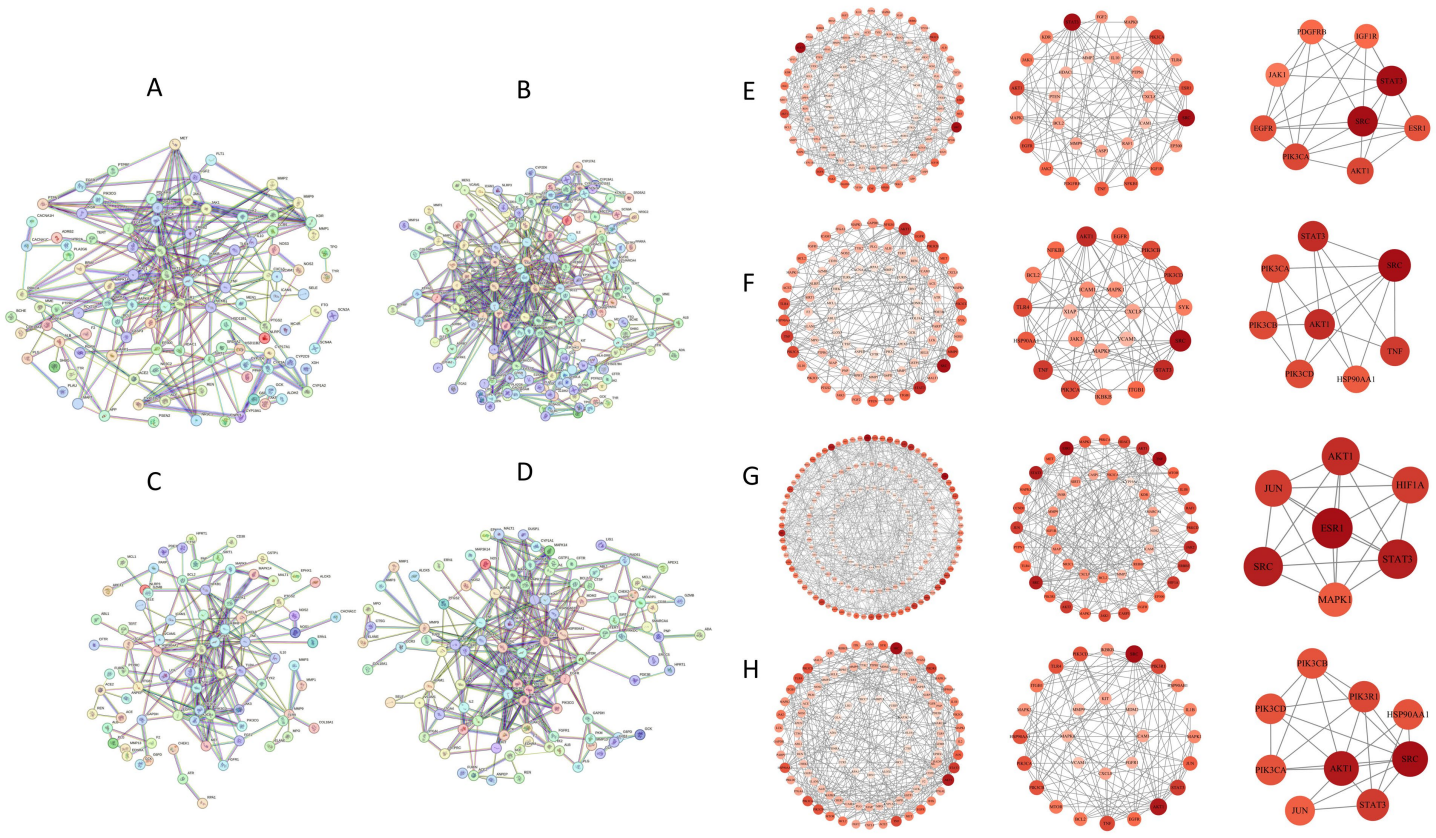
PPI network, topological analysis and core target screening of intersection targets. (**A–D**: STRING-based PPI networks; **E–H**: Cytoscape-based PPI optimization and core target screening. Darker color and larger node area indicate higher connectivity.) **(A)** *Salvia plebeia* active components-T2DM; **(B)** Endophytic bacteria active components-T2DM; **(C)** *Salvia plebeia* active components-BR; **(D)** Endophytic bacteria active components-BR; **(E)** *Salvia plebeia* active components-T2DM; **(F)** Endophytic bacteria active components-T2DM; **(G)** *Salvia plebeia* active components-BR; **(H)** Endophytic bacteria active components-BR.

The overlapping potential targets between components of *Salvia plebeia* and T2DM (144 targets), components of endophytic bacteria and T2DM (210 targets), components of *Salvia plebeia* and BR (95 targets), as well as components of endophytic bacteria and BR (129 targets) were imported into the STRING database to construct the initial protein–protein interaction (PPI) networks. The results showed that the PPI network of *Salvia plebeia* components against T2DM consisted of 105 nodes and 267 edges (Figure 6A). The PPI network of endophytic bacterial components against T2DM contained 160 nodes and 444 edges (Figure 6B). The PPI network of *Salvia plebeia* components against BR included 80 nodes and 190 edges (Figure 6C). The PPI network of endophytic bacterial components against BR comprised 102 nodes and 297 edges (Figure 6D).

Cytoscape 3.9.1 was used for visual optimization of the initial networks. The median values of topological indicators, including betweenness centrality, closeness centrality, degree, eigenvector centrality and local average connectivity (LAC), were set as thresholds to screen core targets. The core targets of *Salvia plebeia* components acting on T2DM were IGF1R, STAT3, JAK1, SRC, ESR1, AKT1, PIK3CA, EGFR and PDGFRB (Figure 6E). The core targets of endophytic bacterial components acting on T2DM were ESR1, MAPK1, STAT3, HIF1A, JUN and SRC (Figure 6F). The core targets of *Salvia plebeia* components acting on BR were AKT1, PIK3CB, PIK3CA, PIK3CD, TNF, HSP90AA1, SRC and STAT3 (Figure 6G). The core targets of endophytic bacterial components acting on BR were PIK3CD, AKT1, STAT3, JUN (Jun proto-oncogene), HSP90AA1, PIK3CB, PIK3CA, PIK3R1 and SRC (Figure 6H).

Nodes were colored with a gradient according to their degree values. Nodes with higher degree values were displayed in darker colors, while those with lower degree values were in lighter colors, which reflected the importance of each target in the corresponding disease.

#### 3.5.3 GO and KEGG pathway enrichment analyses

**Figure 7:**
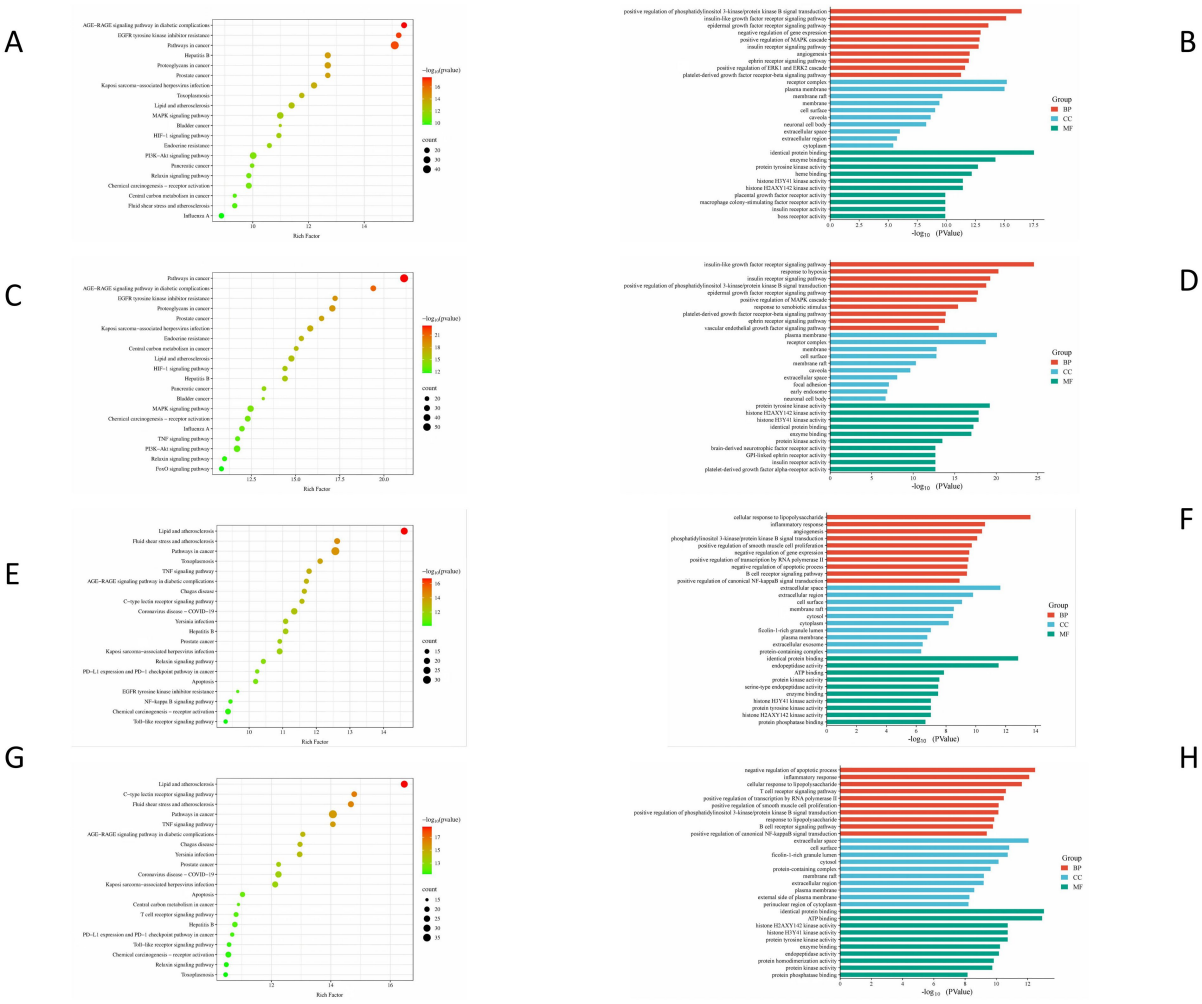
Enrichment analysis of intersection targets between components and diseases. **(A)** KEGG enrichment analysis diagram of intersection targets between *Salvia plebeia* components and T2DM; **(B)** GO enrichment analysis diagram of intersection targets between *Salvia plebeia* components and T2DM; **(C)** KEGG enrichment analysis diagram of intersection targets between endophytic bacterial components and T2DM; **(D)** GO enrichment analysis diagram of intersection targets between endophytic bacterial components and T2DM; **(E)** KEGG enrichment analysis diagram of intersection targets between *Salvia plebeia* components and BR; **(F)** GO enrichment analysis diagram of intersection targets between *Salvia plebeia* components and BR; **(G)** KEGG enrichment analysis diagram of intersection targets between endophytic bacterial components and BR; **(H)** GO enrichment analysis diagram of intersection targets between endophytic bacterial components and BR.

Intersection targets were subjected to KEGG pathway and GO enrichment analyses via the DAVID database (*p* < 0.05). The top 20 pathways and top 10 GO clusters with the smallest *p*-values were selected (Figure 7). Figures 7A,B: for *S. plebeia*-T2DM targets, the most significant KEGG pathway was AGE-RAGE; GO terms included Akt signaling and EGFR signaling. Figures 7C,D: for endophyte-T2DM targets, the top KEGG pathway was Pathways in cancer, followed by AGE-RAGE; GO terms involved hypoxia response and insulin signaling. Figures 7E,F: for *S. plebeia*-BR targets, the most significant KEGG pathway was Lipid and atherosclerosis; GO terms included inflammation and angiogenesis. Figures 7G,H: for endophyte-BR targets, the top KEGG pathway was lipid and atherosclerosis; GO terms involved anti-apoptosis and inflammation. *S. plebeia* and its endophytes act synergistically rather than independently: shared pathways anchor the disease core, while specific pathways supplement regulatory dimensions. T2DM: both target AGE-RAGE, EGFR-TKI resistance, and cancer pathways, regulating AGE and proliferation signaling. BR: Both enrich in lipid/atherosclerosis, fluid shear stress, and TNF pathways, with anti-inflammation and metabolic regulation as core mechanisms. Highly overlapping GO functions and targets suggest endophytes serve as a mini metabolic reservoir for the host. They produce similar bioactive compounds (flavonoids, alkaloids) and enhance therapeutic effects against T2DM and BR via host–microbe interactions.

#### 3.5.4 Construction and analysis of the component-target-disease (C–T–D) network model

**Figure 8:**
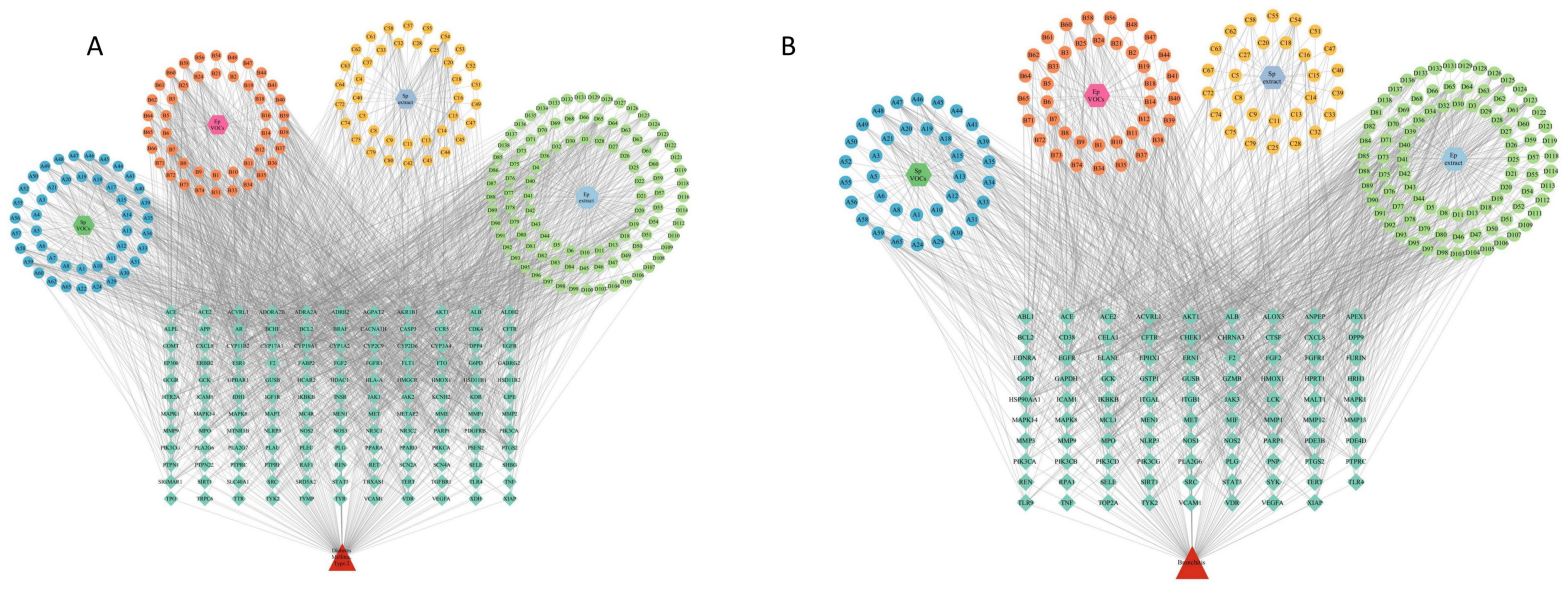
Component-target-disease network diagram (C-T-D). **(A)**: component-target-T2DM network diagram; **(B)** component-target-BR network diagram. [Circular nodes represent active components (blue for volatile components of *Salvia plebeia*, orange for volatile components of endophytic bacteria, yellow for extract components of *Salvia plebeia*, and green for extract components of endophytic bacteria); pentagonal nodes represent component sources; rhombic nodes represent common targets; and triangular nodes represent diseases].

The C–T–D interaction network was constructed using Cytoscape 3.9.1 (Figure 8). For type 2 diabetes mellitus (T2DM) (Figure 8A), the network contained 366 nodes and 2,106 edges. Based on Degree values (indicating node connectivity), the top 5 core components for each source were: SP VOCs (butyl 4-aminobenzoate, etc., Degree 25–21), Ep VOCs (ethyl 3-hydroxyhexanoate, etc., Degree 32–28), SP extract (n-octyl butyrate, etc., Degree 28–14), and Ep extract (2-amino-4-picoline, etc., Degree 29–21). For breast cancer (BR) (Figure 8B), the network had 287 nodes and 1,316 edges. The top 5 core components were: SP VOCs (cyclo(L-Pro-L-Val), etc., Degree 20–11), Ep VOCs (ethyl 3-hydroxyhexanoate, etc., Degree 21–16), SP extract (n-octyl butyrate, etc., Degree 14–5), and Ep extract (4-nitroquinoline N-oxide, etc., Degree 26–19). Degree value rankings for T2DM targets were Ep VOCs > Ep extract > SP extract > SP VOCs; for BR targets, Ep extract > SP extract > Ep VOCs > SP VOCs. These indicate endophytic bacteria may produce more effective metabolites than their host plant.

#### 3.5.5 Construction and analysis of the target–pathway (T–P) network

**Figure 9:**
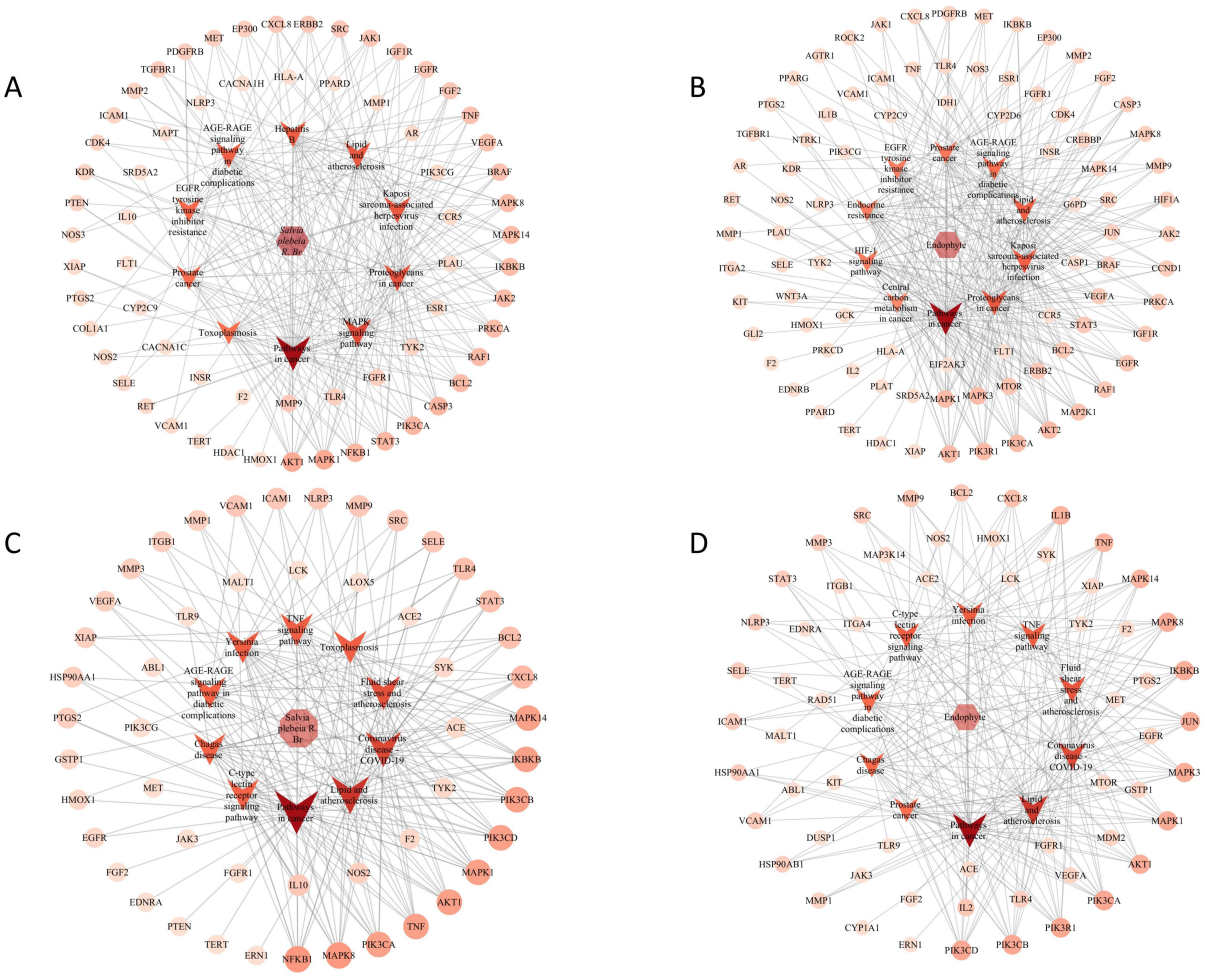
Target–pathway network diagram (T–P) **(A)**: T–P network of *Salvia plebeia* components and T2DM; **(B)** T–P network of endophytic bacterial components and T2DM; **(C)** T–P network of *Salvia plebeia* components and BR; **(D)** T–P network of endophytic bacterial components and BR. (Circular nodes represent targets, V-shaped nodes represent pathways, and the darker the color, the stronger the correlation based on topological analysis).

Based on *p*-values, ten highly relevant signaling pathways were screened. A target–pathway network was subsequently constructed using Cytoscape software. The prediction results indicated that the active components of *Salvia plebeia* exerted effects on type 2 diabetes mellitus mainly through cancer-related pathways, MAPK signaling pathway, AGE-RAGE signaling pathway and others, with *AKT1*, *MAPK1*, *CASP3*, *STAT3* and *NFKB1* identified as the core targets (Figure 9A). The active ingredients of endophytic bacteria regulated type 2 diabetes mellitus via cancer pathways, lipid and atherosclerosis pathway, HIF-1 signaling pathway and related pathways, and the key targets included *MAPK3*, *MAPK1*, *PIK3R1*, *PIK3CA* and *AKT1* (Figure 9B). In the intervention of bronchitis, the active components of *Salvia plebeia* functioned through cancer pathways, atherosclerosis pathway, TNF signaling pathway and so on, involving the targets *MAPK8*, *NFKB1*, *AKT1*, *IKBKB* and *MAPK1* (Figure 9C). Endophytic bacteria mainly exerted anti-bronchitis activities via cancer pathways, inflammatory pathways and metabolic pathways, and the pivotal targets were *PIK3* family genes and *JUN* (Figure 9D).

#### 3.5.6 Molecular docking

The median values of topological indices including Betweenness Centrality, Closeness Centrality, Degree, Eigenvector Centrality, Local Average Connectivity (LAC) and node network indices were set as thresholds to screen the core components and core therapeutic targets for T2DM. Table 1 presents the intersection of core therapeutic targets of *S. plebeia* components and endophytic bacterial components for T2DM, with 4 overlapping core therapeutic targets identified: *ESR1* (estrogen receptor 1), *AKT1* (RAC-alpha serine/threonine-protein kinase), *SRC* (SRC proto-oncogene tyrosine kinase), and *STAT3* (signal transducer and activator of transcription 3). The main components corresponding to *ESR1* included isoborneol (A3), ferruginol (C8), γ-terpineol (B16), fenspiride (D87) and others; those corresponding to *AKT1* comprised cyclic adenosine monophosphate (cAMP, A30), 3,5-di-tert-butylsalicylaldehyde (C32), 2,4-di-tert-butylphenol (B8), dimethocaine (D89); the key components for *SRC* included 8-hydroxyquinoline N-oxide (A58), ferruginol (C8), ethyl salicylate (B40), L-serine (D66); and the major components targeting *STAT3* were octyl acetate (A58), benzyl cinnamate (B61), N,N′-carbonyldiimidazole (D69) and others.

**Table 1:** Screening of core components of *Salvia plebeia* and its endophytic bacteria, and core targets of T2DM.

SP’s component	EP’s component	Target	Betweenness (SP/EP)	Closeness (SP/EP)	Degree (SP/EP)	Eigenvector (SP/EP)	LAC (SP/EP)	Network (SP/EP)
A3 A6 A7 C8 C9 C32 C80	B1 B6 B8 B16 B24 B33 B36 B37 B48 B60 B64 B66 B72 B73 D60 D66 D73 D78 D87 D112 D126	ESR1	30.13, 18.21	0.62, 0.93	11, 13	0.26, 0.35	4.73, 6.92	7.04, 12.28
A30 C32 C58	B3 B8 B39 D65 D82 D89 D95	AKT1	66.95, 11.81	0.64, 0.82	12, 11	0.24, 0.32	3.67, 6.36	6.56, 9.27
A30 A52 C8 C33	B3 B5 B40 B73 D36 D43 D54 D66 D69 D73 D78 D80 D95 D98 D126	SRC	127.53, 16.15	0.72, 0.88	17, 12	0.36, 0.33	5.06, 6.17	11.72, 10.24
A58	B61 D19 D27 D40 D69 D82 D123	STAT3	142.7, 15.92	0.72, 0.88	17, 12	0.34, 0.33	4.82, 6.5	11.41, 10.43

*Salvia plebeia* represents Hama grass and EP represents endophytic bacteria.

Table 2 shows intersection of core therapeutic targets of *S. plebeia* components and endophytic bacterial components for BR, with 4 overlapping core therapeutic targets identified: *PIK3CA* (phosphatidylinositol 3-kinase catalytic subunit alpha), *AKT1* (RAC-alpha serine/threonine-protein kinase), *SRC* (SRC proto-oncogene tyrosine kinase), and *STAT3* (signal transducer and activator of transcription 3). The main components corresponding to *PIK3CA* included cis-3-hexenyl isovalerate (A59), pimpinellin (C58), hydrastine (B58), 8-hydroxyquinoline N-oxide (D77) and others. Among the core therapeutic targets for both T2DM and BR corresponding to the components, 3 targets were overlapping, with consistent corresponding components for these shared targets. The screened targets and the identified components of *S. plebeia* and its endophytic bacteria were subsequently utilized for molecular docking analysis.

**Table 2:** Screening of core components of *Salvia plebeia* and its endophytic bacteria, and core targets of BR.

SP’s component	EP’s component	Target	Betweenness (SP/EP)	Closeness (SP/EP)	Degree (SP/EP)	Eigenvector (SP/EP)	LAC (SP/EP)	Network (SP/EP)
A59 C58	B3 B38 B39 B58 B62 B65 B74 D77 D95 D123 D133	PIK3CA	9.97, 20.93	0.64, 0.63	9, 11	0.28, 0.24	4.89, 5.82	6.43, 8.06
A30 C32 C58	B3 B8 B39 D65 D82 D89 D95	AKT1	39.82, 86.63	0.68, 0.71	11, 16	0.3, 0.33	4, 5.88	6.98, 11.76
A30 A52 C8 C33	B3 B5 B40 B73 D36 D43 D54 D66 D69 D73 D78 D80 D85 D95 D98 D126	SRC	48.5, 101.86	0.72, 0.73	13, 17	0.36, 0.34	4.77, 5.65	8.8, 11.53
A58	B61 D19 D27 D40 D69 D82 D123	STAT3	46.79, 60.11	0.68, 0.66	11, 13	0.28, 0.26	3.45, 4.62	6.26, 7.46

SP represents Hama grass and EP represents endophytic bacteria.

The 2D structural files of bioactive components (small-molecule ligands) and disease therapeutic targets (protein receptors) were imported into the CB-Dock2 web server. Since each small-molecule ligand binds to the protein receptor at multiple sites, the conformational data with the minimum binding energy (highest affinity) was selected for analysis in this study. A lower binding energy value indicates a stronger binding capacity between the ligand and the receptor.

A comparison of the binding energies between small-molecule ligands derived from *S. plebeia* and its endophytic bacteria and their corresponding protein receptors was conducted. As shown in Table 3, the protein receptor *ESR1* exhibited the strongest binding affinity with ligand C8 (from *S. plebeia*) and ligand B72 (from endophytic bacteria); notably, the endophytic bacterial small-molecule ligand had the lowest binding energy value, with a stronger binding capacity than that of the *S. plebeia*-derived small-molecule ligand. The protein receptor *AKT1* showed the strongest binding affinity with ligand A30 (from *S. plebeia*) and ligand D65 (from endophytic bacteria); *SRC* exhibited the strongest binding affinity with ligand A30 (from *S. plebeia*) and ligand B73 (from endophytic bacteria); *STAT3* had the strongest binding affinity with ligand A58 (from *S. plebeia*) and ligand D123 (from endophytic bacteria); and *PIK3CA* displayed the strongest binding affinity with ligand C58 (from *S. plebeia*) and ligand B74 (from endophytic bacteria).

**Table 3:** Binding energy of molecular docking between core components of *Salvia plebeia* and its endophytic Bacteria, and core targets.

ESR1	AKT1	SRC	STAT3	PIK3CA
A3(−5.7)	A30(−6)	A30(−7.6)	A58(−5.0)	A59(−72.2)
A6(−5.8)	C32(−5.8)	A52/C33(−6.8)	D19(−4.1)	C58(−80.8)
A7(−6.7)	C58(−5.7)	C8(−7.5)	D27(−5.1)	B3(−73.4)
C8(−8.6)	B3(−4.4)	B3(−4.9)	D40(−5.4)	B38(−69.8)
C9(−8.0)	B8(−5.0)	B5(−5.1)	D69(−5.7)	B39(−62.4)
C32(−7.5)	B39(−4.3)	B40(−6.1)	D82(−5.4)	B58(−106.2)
B1(−6.2)	D65(−5.1)	B73(−7.5)	D123(−6.3)	B62(−73.2)
B6(−5.8)	D82(−4.4)	D36(−6.3)		B65(−101.1)
B8(−7.0)	D95(−4.6)	D43(−4.6)		B74(−124.1)
B16(−6.2)		D54(−6.8)		D77(−80.6)
B24(−5.8)		D66(−7.2)		D95(−76.4)
B33(−6.7)		D69(−5.2)		D123(−77.1)
B36(−5.3)		D73(−5.3)		D133(−53.7)
B37(−5.7)		D78(−5.7)		
B48(−7.1)		D80(−6.1)		
B60(−4.9)		D95(−5.3)		
B64(−5.1)		D98(−4.3)		
B66(−8.1)		D126(−5.4)		
B72(−8.9)				
B73(−8.4)				
D60(−7.9)				
D66(−8.4)				
D73(−5.8)				
D78(−6.7)				
D87(−5.7)				
D112(−6.3)				
D126(−8.5)				

The core components of *Salvia plebeia* and endophytic bacteria that exhibit strong binding ability to targets are marked in red shades.

**Figure 10:**
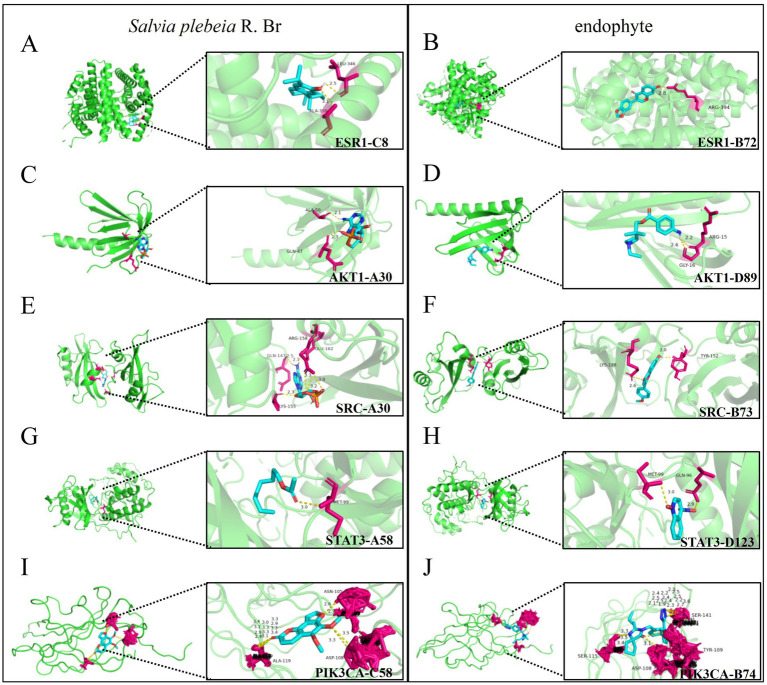
Molecular docking between active components and effective targets **(A)**: Molecular docking of target ESR1 and component C8; **(B)**: Molecular docking of target ESR1 and component B72; **(C)**: Molecular docking of target AKT1 and component A30; **(D)**: Molecular docking of target AKT1 and component D89; **(E)**: Molecular docking of target SRC and component A30; **(F)**: Molecular docking of target SRC and component A73; **(G)**: Molecular docking of target STAT3 and component A58; **(H)**: Molecular docking of target STAT3 and component D123; **(I)**: Molecular docking of target PIK3CA and component C58; **(J)**: Molecular docking of target PIK3CA and component B74. (Green represents protein receptors; light blue represents small molecule ligands; pink represents bound protein residues).

PyMOL software was employed for the visualization analysis of the optimal binding conformations (with the minimum binding energy) between core components and core targets. As shown in Figure 10, some protein residues were involved in the binding of core components to core targets and their corresponding hydrogen bond lengths. For target *ESR1*, protein residues (hydrogen bond lengths) involved in its binding to *S. plebeia* component C8 were LEU-346 (2.5 Å) and ALA-350 (3.1 Å), while those for its binding to endophytic bacterial component B72 were ARG-394 (2.8 Å). For target *AKT1*, protein residues (hydrogen bond lengths) involved in its binding to *S. plebeia* component A30 were ALA-50 (2.1 Å) and GLN-47 (2.3 Å), whereas those for its binding to endophytic bacterial component D89 were ARG-15 (2.2 Å) and GLY-16 (2.6 Å). For target *SRC*, protein residues (hydrogen bond lengths) involved in its binding to *S. plebeia* component A30 were ARG-158 (3.3 Å, 3.2 Å, 3.0 Å), GLU-162 (2.1 Å), GLN-147 (2.5 Å) and LYS-155 (2.3 Å); protein residues (hydrogen bond lengths) for its binding to endophytic bacterial component B73 were consistent with the above, including ARG-158 (3.3 Å, 3.2 Å, 3.0 Å), GLU-162 (2.1 Å), GLN-147 (2.5 Å) and LYS-155 (2.3 Å). For target *PIK3CA*, protein residues (hydrogen bond lengths) involved in its binding to *S. plebeia* component C58 were ASN-105 (2.6 Å, 2.7 Å), ASP-108 (3.3 Å, 3.5 Å) and ALA-119 (3.4 Å, 3.1 Å, 2.9 Å, 2.9 Å, 3.0 Å, 3.3 Å, 3.3 Å, 3.1 Å, 3.3 Å, 2.9 Å, 3.3 Å, 3.4 Å); those for its binding to endophytic bacterial component B73 were TYR-109 (3.4 Å), ASP-108 (3.1 Å), SER-115 (3.3 Å, 3.5 Å, 3.4 Å) and SER-141 (2.4 Å, 2.5 Å, 2.1 Å, 2.2 Å, 2.4 Å, 2.5 Å, 2.4 Å, 1.9 Å, 2.3 Å, 2.5 Å, 2.5 Å, 2.5 Å, 2.2 Å, 3.6 Å, 2.7 Å).

## 4 Discussion

Most previous relevant researches merely focused on single bioactivity screening of medicinal plant endophytes, predominantly exploring endophyte-mediated regulation on host secondary metabolite biosynthesis and screening endophytic strains capable of producing anti-tumor and anti-infective metabolites; systematic investigations regarding synergistic pharmacological mechanisms driven by host–endophyte interactions remain scarce (Zotchev, 2024; Yu et al., 2024). Distinct from prior studies, the present work innovatively adopted network pharmacology to predict correlations between secondary metabolites derived from host plant and endophytes, and further evaluated stable binding affinity between core bioactive constituents and disease-related targets at the molecular level. This research provides novel insights for investigations on symbiotic metabolic crosstalk and synergistic pharmacodynamic effects of *S. plebeia*–endophyte consortia.

Endophytic bacteria were isolated from various tissues of *S. plebeia*, and three typical phytopathogenic fungi including *Magnaporthe oryzae*, *Fusarium oxysporum* and *Fusarium graminearum* were selected as indicator pathogens for primary antifungal bioassay. Six dominant strains (L1, L24, R86, R109, S10, S26) with mycelial inhibition rates exceeding 50% were screened out. Both fermentation broths and volatile organic compounds (VOCs) generated by these prioritized isolates significantly suppressed mycelial growth of tested phytopathogens (Figures 2A–C). Additional screening yielded endophytic isolates with remarkable inhibitory activities against *Escherichia coli*, *Bacillus subtilis* and *Staphylococcus aureus*, alongside evident antioxidant capacities (Figures 4C, E, F). These results demonstrated that secondary metabolites synthesized by antagonistic endophytes from *S. plebeia* contain abundant antimicrobial ingredients. The antifungal potency against phytopathogens was closely associated with VOCs released by *S. plebeia*-derived endophytic bacteria; specifically, VOCs from the six high-efficiency strains exerted robust inhibitory effects on *M. oryzae*, *F. oxysporum* and *F. graminearum*. Headspace solid-phase microextraction coupled with gas chromatography–mass spectrometry (HS-SPME-GC–MS) profiling identified multiple VOC constituents with verified antagonistic activities against the three target phytopathogens (Supplementary Table S3): propyl salicylate inhibits *M. oryzae*, guaiacol suppresses *F. oxysporum*, and γ-terpinene displays bioactivity against *F. graminearum* (Patel et al., 2021; Gao et al., 2021; Moumni et al., 2021). Collectively, VOCs from *S. plebeia* endophytes are rich in bioactive antimicrobials with promising broad-spectrum antimicrobial potential, furnishing direct material evidence for further research on the biocontrol mechanisms of endophytic VOCs from *S. plebeia*. Consistently, numerous previous studies have retrieved antimicrobial endophytes from medicinal plants. For instance, endophytic bacteria isolated from *Glycyrrhiza uralensis* exhibited broad-spectrum antagonism against various human and plant pathogens upon *in vitro* bioassays; specifically, *Bacillus atrophaeus* XEGI50 was validated to synthesize pathogen-specific antimicrobial metabolites during co-cultivation with target microbes (Mohamad et al., 2018). Endophytes recovered from *Origanum heracleoticum* L. displayed differentiated broad-spectrum antagonism against multidrug-resistant human pathogenic bacteria, and several isolates efficiently produced characteristic volatile metabolites (Semenzato et al., 2024). Our findings further confirm that endophytic bacteria residing in *S. plebeia* serve as promising reservoirs for novel antimicrobial lead compounds, reinforcing the consensus that endophytes associated with medicinal plants constitute an invaluable resource to discover unprecedented bioactive antimicrobials.

Based on 16S rRNA gene sequencing and phylogenetic analysis (Figure 3), the 10 functionally characterized endophytic isolates were taxonomically affiliated to three genera: *Bacillus*, *Pseudomonas* and *Enterobacter*. Cumulative literature has documented that strains belonging to these three genera commonly possess multiple beneficial traits including phosphate solubilization, plant-growth-promoting substance biosynthesis, functional enzyme secretion and phytopathogen antagonism (Ngalimat et al., 2021; Murugappan et al., 2013). The functional phenotypes of strains tested herein are consistent with published findings, verifying the reliability of our experimental data.

Published literatures have proven that the metabolic crosstalk between secondary metabolites of host plants and their resident endophytes constitutes a sophisticated dynamic process, wherein endophyte-derived metabolites interact with host secondary products to generate novel bioactive molecules. For example, plant-microbiota symbiosis improves biomass accumulation of *Salvia miltiorrhiza*, and host-specific endophytic consortia modulate the biosynthesis of tanshinone-type characteristic bioactive compounds (Pandey et al., 2023). Building on these advances, the current study further explored symbiotic interaction mechanisms between medicinal host and endophytes via gas chromatography–mass spectrometry (GC–MS), which systematically profiled shared and differential metabolites of *S. plebeia* and its associated endophytes. Analogous to endophytic fungi of *Taxus* spp. capable of paclitaxel biosynthesis, endophytes isolated from *S. plebeia* represent an untapped treasure trove of bioactive metabolites (Qian et al., 2016). Prior network topology quantification on herbal constituent-target interactions validated that clinically compatible herbal formulae possess superior network topological proximity relative to random herbal combinations, which scientifically elucidates synergistic mechanisms underlying traditional Chinese medicinal compatibility (Wang et al., 2021). Using network pharmacology as a core analytical framework, the present research unravelled intrinsic correlations and interactive rules between metabolites originating from host *S. plebeia* and its endophytic bacteria. Remarkable overlap was observed in core signaling pathways and Gene Ontology (GO) functional annotations targeted by metabolites from host plant and endophytes (Figure 7), implying that endophytes may function as an in planta miniature metabolic reservoir that participates in host pharmacodynamic compound biosynthesis via sharing core metabolic pathways and synergistically regulating host metabolism and immune responses; follow-up co-cultivation trials are required to validate this hypothesis. Node degree calculation of the constituent-target-disease network (Figures 8, 9) indicated that metabolites derived from endophytes displayed higher overall network connectivity and stronger regulatory effects toward the two investigated diseases compared with intrinsic phytochemicals of *S. plebeia*, and volatile metabolites contributed the most prominent regulatory potency, further supporting that medicinal plant endophytes are elite candidates to mine novel antimicrobial agents (Cui et al., 2026). Five hub targets shared by host phytochemicals and endophytic metabolites against the two diseases were screened: *ESR1*, *AKT1*, *SRC*, *STAT3* and *PIK3CA*(Figure 10). Molecular docking results revealed that endophyte-originated bioactive compounds exhibited superior binding affinities against these core targets relative to native metabolites of *S. plebeia*; several endophytic constituents formed stable hydrogen bonds with target proteins and shared identical binding amino acid residues with host-derived phytochemicals at binding pockets. These molecular docking outcomes provide molecular-level evidence for synergistic pharmacological potential between *S. plebeia* and its endophytes, yet *in vivo* and *in vitro* functional verification assays are still essential to decipher detailed underlying pharmacodynamic mechanisms.

Future research will prioritize functional validation of core bioactive metabolites, characterize key biosynthetic gene clusters and regulatory networks governing signature metabolite production in dominant endophytes, and optimize submerged fermentation and VOC-inducing culture protocols for prioritized strains. Moreover, subsequent investigations will explore the industrial application potential of these functional endophytes in multiple fields including eco-friendly agricultural biocontrol, natural antioxidant formulation manufacturing, and adjuvant therapy for inflammatory and metabolic disorders, facilitating high-value exploitation and industrial transformation of *S. plebeia*-associated endophytic microbial resources.

## 5 Conclusion

In this study, endophytic bacteria with diverse functions were isolated from *S. plebeia* R. Br. All strains exhibited enzyme-producing, growth-promoting and stress-resistant properties with varying activities. Six broad-spectrum antifungal strains were screened, and their fermentation broths and volatile components showed remarkable antagonistic effects against pathogenic fungi. Mass spectrometry analysis confirmed the presence of common bioactive constituents in *S. plebeia* and its endophytes. For the first time, this study combined network pharmacology and molecular docking to predict the synergistic mechanism, core active ingredients, key targets and disease-related pathways of the two. It should be noted that the above mechanistic results are merely bioinformatic predictions and have not been verified by in vitro and in vivo experiments. The molecular interaction mechanism remains to be further explored. Follow-up research will focus on validating core active components, elucidating their synthetic pathways and optimizing the culture conditions of functional strains, so as to facilitate the practical application of these microbial resources.

## Data Availability

The datasets presented in this study can be found in online repositories. The names of the repository/repositories and accession number(s) can be found in the article/supplementary material.

## Footnotes

1
https://pubchem.ncbi.nlm.nih.gov/

2
https://swisstargetprediction.ch/

3
https://www.genecards.org/

4
https://www.omim.org/

5
https://ttd.idrblab.cn/

6
https://cn.string-db.org/

7
https://davidbioinformatics.nih.gov/

8
https://www.uniprot.org/

9
https://cadd.labshare.cn/cb-dock2/php/index.php
