# Supplementary material for: Network pharmacological prediction on metabolites of dominant endophytic strains from Salvia plebeia R. Br
Source: Front Microbiol. 2026 Jun 29;17:1847318. doi: 10.3389/fmicb.2026.1847318 (PMC13357940; doi:10.3389/fmicb.2026.1847318)
Supplement: Supplementary file 1 [file Supplementary_file_1.DOCX]

**Supplementary Materials**

**1 Supplementary Figures and Tables**

**1.1 Sampling locations of *Salvia plebeia***

Salvia plebeia R. Br. (common name: Hama-cao, Lizhi-cao) is an annual or biennial herb of the Lamiaceae family. Plants are 15-90 cm tall, with erect, quadrangular, branched stems covered with grayish-white downward-directed pubescence. Leaves are simple, opposite, elliptic-ovate to elliptic-lanceolate, 2-6 cm long and 0.8-2.5 cm wide, with rounded or cuneate bases, obtuse or acute apices, and crenate-serrate margins. The leaf surface is markedly wrinkled, with sparse stiff hairs above, short pubescence beneath, and scattered yellowish-brown glandular dots. Verticillasters are 2-6-flowered, arranged in terminal or axillary racemose panicles; corollas are bilabiate, pale purple to bluish-purple, 4.5-6 mm long. Nutlets are obovoid, brownish, and glandular.

Table S1 Sampling information of *Salvia plebeia* samples

| **Number** | **Site** | **Latitude(N)** | **Longitude(E)** | **Altitude/m** |
| --- | --- | --- | --- | --- |
| 1 | Puyang, Henan province | 36°2′10″ | 115°19′12″ | 45 |
| 2 | Pingdingshan, Henan province | 34°4′55″ | 112°49′52″ | 452 |
| 3 | Shangqiu, Henan province | 34°29′2″ | 115°27′56″ | 51 |
| 4 | Xuchang, Henan province | 33°26′52″ | 112°55′31″ | 79 |
| 5 | Nanyang, Henan province | 34°5′10″ | 113°49′18″ | 162 |
| 6 | Zhumadian, Henan province | 33°17′41″ | 113°39′22″ | 64 |
| 7 | Xinxiang, Henan province | 35°28′37″ | 133°46′12″ | 1150 |
| 8 | Jining, Shandong province | 35°25′27″ | 116°0′46″ | 40 |
| 9 | Xuzhou, Jiangsu province | 34°31′19″ | 118°6′45″ | 24 |

**1.2 Identification of active components in *Salvia plebeia* and its endophytes**

The data on the endophytic bacterial community and host plant chemical composition analyzed in this study are presented in large multi-page tables. Due to formatting constraints, these tables are provided as Supplementary Materials and can be found therein.

Table S2 Composition and relative content of volatile organic compounds in roots and leaves of *Salvia plebeia*

| No. | Components | CAS | Molecular formula | Retention Time/min | Relative content/% | |
| --- | --- | --- | --- | --- | --- | --- |
|  |  |  |  |  | Root | Leaf |
| A1 | Furan, 2-pentyl- | 3777-69-3 | C_9_ H_14_ O | 9.425 | 4.45 | - |
| A2 | 5-(p-Tolyl)-1H-tetrazole | 24994-04-5 | C_8_ H_8_ N_4_ | 12.294 | 0.73 | - |
| A3 | Isoborneol | 124-76-5 | C_10_ H_18_ O | 14.958 | 14.21 | - |
| A4 | Benzene, 1-methyl-3-(1-methylethyl)- | 535-77-3 | C_10_ H_14_ | 10.332 | 10.17 | - |
| A5 | Camphor | 76-22-2 | C_10_ H_16_ O | 14.222 | 6.45 | - |
| A6 | endo-Borneol | 507-70-0 | C_10_ H_18_ O | 14.955 | 25.22 | - |
| A7 | Azulene | 275-51-4 | C_10_ H_8_ | 15.538 | 0.74 | 0.22 |
| A8 | 1,3-Cyclohexadiene, 1-methyl-4-(1-methylethyl)- | 99-86-5 | C_10_ H_16_ | 17.269 | 0.69 | 0.01 |
| A9 | Benzene, 1,3-dimethyl- | 108-38-3 | C_8_ H_10_ | 6.67 | 0.29 | 0.48 |
| A10 | Valproic Acid | 99-66-1 | C_8_ H_16_ O_2_ | 9.351 | 0.26 | - |
| A11 | Benzene, 1-methoxy-4-methyl- | 104-93-8 | C_8_ H_10_ O | 9.943 | 1.80 | - |
| A12 | Ethanone, 1-(3-hydroxyphenyl)- | 121-71-1 | C_8_ H_8_ O_2_ | 12.27 | 0.21 | - |
| A13 | Benzoic acid, 2,3-dihydroxy- | 303-38-8 | C_7_ H_6_ O_4_ | 15.402 | 0.76 | - |
| A14 | Eucalyptol | 470-82-6 | C_10_ H_18_ O | 10.575 | 2.35 | - |
| A15 | Benzene, 1,4-dimethoxy- | 150-78-7 | C_8_ H_10_ O_2_ | 14.275 | 1.00 | - |
| A16 | 1,2-Benzenedicarbonitrile | 91-15-6 | C_8_ H_4_ N_2_ | 15.528 | 0.30 | - |
| A17 | Nitroguanidine | 556-88-7 | C H_4_ N_4_ O_2_ | 15.656 | 0.34 | - |
| A18 | N-Acetyltyramine | 1202-66-0 | C_10_ H_13_ N O_2_ | 8.379 | 0.04 | - |
| A19 | Bicyclo[2.2.1]heptan-2-ol, 1,7,7-trimethyl-, (1S-endo)- | 464-45-9 | C_10_ H_18_ O | 14.954 | 21.76 | - |
| A20 | Thymol | 89-83-8 | C_10_ H_14_ O | 20.129 | 0.05 | - |
| A21 | 10(9H)-Acridineacetic acid, 9-oxo- | 38609-97-1 | C_15_ H_11_ N O_3_ | 48.246 | 0.05 | - |
| A22 | Heptanal | 111-71-7 | C_7_ H_14_ O | 7.368 | 0.27 | - |
| A23 | Dimethyl (1-diazo-2-oxopropyl)phosphonate | 90965-06-3 | C_5_ H_9_ N_2_ O_4_ P | 24.372 | 0.73 | 0.08 |
| A24 | Benzene, 1-(1,5-dimethyl-4-hexenyl)-4-methyl- | 644-30-4 | C_15_ H_22_ | 29.151 | 1.57 | 0.29 |
| A25 | 1-Propene, 2-methoxy- | 116-11-0 | C_4_ H_8_ O | 1.994 | 0.46 | - |
| A26 | Propane, 2-isocyano-2-methyl- | 7188-38-7 | C_5_ H_9_ N | 9.029 | 0.11 | 0.03 |
| A27 | 2-Propenoic acid, anhydride | 2051-76-5 | C_6_ H_6_ O_3_ | 10.496 | 0.17 | - |
| A28 | Pyridine, 2-methyl-, 1-oxide | 931-19-1 | C_6_ H_7_ N O | 12.489 | 0.69 | - |
| A29 | Naphthalene | 91-20-3 | C_10_ H_8_ | 15.536 | 0.31 | - |
| A30 | Adenosine 3',5'-cyclic monophosphate | 60-92-4 | C_10_ H_12_ N_5_ O_6_ P | 15.857 | 3.81 | - |
| A31 | Formamide, N-methyl-N-phenyl- | 93-61-8 | C_8_ H_9_ N O | 19.151 | 0.01 | - |
| A32 | Pentylenetetrazol | 54-95-5 | C_6_ H_10_ N_4_ | 6.465 | - | 0.12 |
| A33 | Nonanal | 124-19-6 | C_9_ H_18_ O | 12.772 | - | 1.99 |
| A34 | cis-3-Hexenyl iso-butyrate | 41519-23-7 | C_10_ H_18_ O_2_ | 14.13 | - | 0.16 |
| A35 | Methyl salicylate | 119-36-8 | C_8_ H_8_ O_3_ | 15.976 | - | 1.16 |
| A36 | Hydroxyurea | 127-07-1 | C H_4_ N_2_ O_2_ | 35.515 | - | 0.18 |
| A37 | Propanal, 2-methyl- | 78-84-2 | C_4_ H_8_ O | 1.984 | - | 0.11 |
| A38 | Ethyl isocyanoacetate | 2999-46-4 | C_5_ H_7_ N O_2_ | 5.291 | - | 0.34 |
| A39 | 3-Nonanone | 925-78-0 | C_9_ H_18_ O | 11.374 | - | 0.07 |
| A40 | Octanal | 124-13-0 | C_8_ H_16_ O | 9.745 | - | 0.51 |
| A41 | Phenylethyl Alcohol | 60-12-8 | C_8_ H_10_ O | 13.138 | - | 0.86 |
| A42 | Ethyl diazoacetate | 623-73-4 | C_4_ H_6_ N_2_ O_2_ | 38.393 | - | 0.16 |
| A43 | 2-Propanamine, N,N'-methanetetraylbis- | 693-13-0 | C_7_ H_14_ N_2_ | 32.96 | - | 0.11 |
| A44 | Resorcinol, 2-acetyl- | 699-83-2 | C_8_ H_8_ O_3_ | 16.984 | - | 0.11 |
| A45 | Furoylglycine | 5657-19-2 | C_7_ H_7_ N O_4_ | 24.473 | - | 0.03 |
| A46 | Butamben | 94-25-7 | C_11_ H_15_ N O_2_ | 28.907 | - | 0.01 |
| A47 | Cyclo(L-prolyl-L-valine) | 2854-40-2 | C_10_ H_16_ N_2_ O_2_ | 39.503 | - | 58.45 |
| A48 | Cyclo(Pro-Leu) | 5654-86-4 | C_11_ H_18_ N_2_ O_2_ | 43.261 | - | 25.60 |
| A49 | 2-Butanone, 4-phenyl- | 2550-26-7 | C_10_ H_12_ O | 17.888 | - | 0.40 |
| A50 | Betaine | 107-43-7 | C_5_ H_11_ N O_2_ | 2.721 | - | 0.68 |
| A51 | Benzene, 1,2,4-trimethyl- | 95-63-6 | C_9_ H_12_ | 9.487 | - | 0.02 |
| A52 | 8-Quinolinol, 1-oxide | 1127-45-3 | C_9_ H_7_ N O_2_ | 25.147 | - | 0.07 |
| A53 | Styrene | 100-42-5 | C_8_ H_8_ | 7.108 | - | 0.45 |
| A54 | 3-Pyridinecarboxamide, 1-oxide | 1986-81-8 | C_6_ H_6_ N_2_ O_2_ | 14.286 | - | 0.04 |
| A55 | Phenethyl isocyanate | 1943-82-4 | C_9_ H_9_ N O | 28.241 | - | 0.02 |
| A56 | Bupivacaine | 38396-39-3 | C_18_ H_28_ N_2_ O | 41.512 | - | 0.02 |
| A57 | p-Cymene | 99-87-6 | C_10_ H_14_ | 10.336 | - | 0.05 |
| A58 | Acetic acid, octyl ester | 112-14-1 | C_10_ H_20_ O_2_ | 16.63 | - | 5.75 |
| A59 | n-Valeric acid cis-3-hexenyl ester | 35852-46-1 | C_11_ H_20_ O_2_ | 17.428 | - | 0.23 |
| A60 | 1H-Tetrazole-1-acetic acid | 21732-17-2 | C_3_ H_4_ N_4_ O_2_ | 9.563 | - | 0.49 |
| A61 | 2,5-Furandicarboxaldehyde | 823-82-5 | C_6_ H_4_ O_3_ | 10.604 | - | 0.02 |
| A62 | Pentanoic acid, 2,4-dioxo-, methyl ester | 20577-61-1 | C_6_ H_8_ O_4_ | 17.592 | - | 0.11 |
| A63 | Methanamine, N,N-dimethyl-, N-oxide | 1184-78-7 | C_3_ H_9_ N O | 21.444 | - | 0.46 |
| A64 | 3(2H)-Furanone, 4-hydroxy-5-methyl- | 19322-27-1 | C_5_ H_6_ O_3_ | 34.899 | - | 0.02 |
| A65 | 3-Isopropyl-1,2-benzenediol | 2138-48-9 | C_9_ H_12_ O_2_ | 16.997 | - | 0.10 |

Note: Compounds with a library matching similarity score ≥ 70% were selected as valid identification results, the same below.

Table S3 Composition and relative content of volatile organic compounds from endophytic bacteria of *Salvia plebeia*

| No. | Components | CAS | Molecular formula | Retention Time/min | Relative content/% | | | | | |
| --- | --- | --- | --- | --- | --- | --- | --- | --- | --- | --- |
|  |  |  |  |  | L1 | L24 | R86 | R109 | S10 | S26 |
| B1 | Phenol, 2-ethyl-6-methyl- | 1687-64-5 | C_9_ H_12_ O | 20.492 | 16.98 | - | - | - | - | - |
| B2 | Tetradecanoic acid, ethyl ester | 124-06-1 | C_16_ H_32_ O_2_ | 25.535 | 8.06 | 1.60 | 0.77 | 1.44 | 1.21 | 4.36 |
| B3 | 2-Tetradecanone | 2345-27-9 | C_14_ H_28_ O | 23.915 | 6.31 | 4.52 | - | - | - | - |
| B4 | Phenylethyl Alcohol | 60-12-8 | C_8_ H_10_ O | 18.539 | 8.66 | 2.59 | - | 82.05 | 86.63 | 81.95 |
| B5 | Dodecanoic acid, ethyl ester | 106-33-2 | C_14_ H_28_ O_2_ | 24.17 | 1.23 | 1.71 | 0.18 | 0.30 | 1.07 | 2.82 |
| B6 | 2-Undecanone | 112-12-9 | C_11_ H_22_ O | 20.995 | 3.26 | 26.78 | 23.80 | - | 0.53 | - |
| B7 | Decanoic acid, ethyl ester | 110-38-3 | C_12_ H_24_ O_2_ | 23.052 | 2.70 | 1.46 | 1.16 | 0.20 | - | 2.13 |
| B8 | 2,4-Di-tert-butylphenol | 96-76-4 | C_14_ H_22_ O | 23.524 | 1.96 | 0.27 | 0.64 | 1.26 | 0.72 | 0.80 |
| B9 | Benzeneacetic acid, ethyl ester | 101-97-3 | C_10_ H_12_ O_2_ | 20.289 | 15.26 | 4.55 | 9.26 | - | 2.59 | 2.30 |
| B10 | 2-Nonanone | 821-55-6 | C_9_ H_18_ O | 18.18 | 2.23 | 4.95 | 41.08 | 0.80 | 0.50 | 0.64 |
| B11 | Undecanoic acid, ethyl ester | 627-90-7 | C_13_ H_26_ O_2_ | 24.639 | 3.24 | 0.76 | - | 0.13 | - | - |
| B12 | Heptadecanoic acid, ethyl ester | 14010-23-2 | C_19_ H_38_ O_2_ | 27.574 | 15.98 | 1.30 | - | - | - | - |
| B13 | Propane, 2-isocyanato- | 1795-48-8 | C_4_ H_7_ N O | 18.39 | 1.19 | - | - | 0.19 | 0.84 | - |
| B14 | Octanoic acid, ethyl ester | 106-32-1 | C_10_ H_20_ O_2_ | 19.6 | 0.64 | 0.29 | 3.57 | 0.40 | - | 1.28 |
| B15 | Naphthalene, 1,6-dimethyl- | 575-43-9 | C_12_ H_12_ | 22.714 | 0.22 | - | - | 0.11 | - | - |
| B16 | Cyclohexanol, 1-methyl-4-(1-methylethylidene)- | 586-81-2 | C_10_ H_18_ O | 19.822 | 0.78 | - | - | - | - | - |
| B17 | Propane, 1-isocyanato- | 110-78-1 | C_4_ H_7_ N O | 23.824 | 0.19 | - | - | 4.41 | - | - |
| B18 | Benzeneacetic acid, methyl ester | 101-41-7 | C_9_ H_10_ O_2_ | 19.389 | 0.04 | - | - | - | - | - |
| B19 | 2-Tridecanone | 593-08-8 | C_13_ H_26_ O | 23.36 | 9.92 | 46.10 | 2.56 | - | - | - |
| B20 | Benzofurazan | 273-09-6 | C_6_ H_4_ N_2_ O | 17.223 | 0.13 | - | - | - | - | - |
| B21 | 2-Butenoic acid, butyl ester | 7299-91-4 | C_8_ H_14_ O_2_ | 9.479 | 0.27 | - | - | - | - | - |
| B22 | [2,2-bis(Propan-2-yl)-1,3-dioxolan-4-yl]methanol | 470-43-9 | C_10_ H_20_ O_3_ | 21.42 | 0.74 | - | - | - | - | - |
| B23 | 1,2,4-Benzotriazin-3-amine, 1,4-dioxide | 27314-97-2 | C_7_ H_6_ N_4_ O_2_ | 30.716 | 0.02 | - | - | - | - | - |
| B24 | 2-Decanol | 1120-06-5 | C_10_ H_22_ O | 19.657 | - | 0.21 | - | - | 0.08 | 0.06 |
| B25 | Hexanoic acid, ethyl ester | 123-66-0 | C_8_ H_16_ O_2_ | 16.025 | - | 1.90 | - | - | - | - |
| B26 | Cyclohexane, isocyanato- | 3173-53-3 | C_7_ H_11_ N O | 24.802 | - | 0.18 | - | - | - | 0.12 |
| B27 | Anisole | 100-66-3 | C_7_ H_8_ O | 17.07 | - | 0.03 | - | - | - | - |
| B28 | Nitric acid, ethyl ester | 625-58-1 | C_2_ H_5_ N O_3_ | 2.316 | - | 0.10 | - | - | - | - |
| B29 | Azulene | 275-51-4 | C_10_ H_8_ | 19.447 | - | 0.38 | - | - | - | - |
| B30 | Etilefrine | 709-55-7 | C_10_ H_15_ N O_2_ | 13.488 | - | 0.05 | - | - | - | - |
| B31 | Phenol, 2-methoxy- | 90-05-1 | C_7_ H_8_ O_2_ | 18.114 | - | 0.15 | - | - | - | - |
| B32 | Indene | 95-13-6 | C_9_ H_8_ | 17.118 | - | 0.06 | 0.01 | - | - | - |
| B33 | Ethanone, 1-(2,4,6-trihydroxyphenyl)- | 480-66-0 | C_8_ H_8_ O_4_ | 24.368 | - | 0.01 | - | - | - | - |
| B34 | 10(9H)-Acridineacetic acid, 9-oxo- | 38609-97-1 | C_15_ H_11_ N O_3_ | 34.24 | - | 0.02 | - | - | - | - |
| B35 | Benzoic acid, ethyl ester | 93-89-0 | C_9_ H_10_ O_2_ | 19.277 | - | - | 1.97 | 0.72 | 0.41 | 0.84 |
| B36 | 1-Decanol | 112-30-1 | C_10_ H_22_ O | 20.932 | - | - | 0.27 | - | - | - |
| B37 | 2-Undecanol | 1653-30-1 | C_11_ H_24_ O | 21.095 | - | - | 8.54 | - | - | - |
| B38 | Ethyl (E)-2-octenoate | 7367-82-0 | C_10_ H_18_ O_2_ | 20.268 | - | - | 2.61 | - | - | - |
| B39 | Hexanoic acid, 3-hydroxy-, ethyl ester | 2305-25-1 | C_8_ H_16_ O_3_ | 18.723 | - | - | 0.59 | - | - | - |
| B40 | Benzoic acid, 2-hydroxy-, ethyl ester | 118-61-6 | C_9_ H_10_ O_3_ | 20.705 | - | - | 1.18 | - | - | - |
| B41 | Geranyl formate | 105-86-2 | C_11_ H_18_ O_2_ | 22.417 | - | - | 1.61 | - | - | - |
| B42 | 2-Furancarboxylic acid, ethyl ester | 614-99-3 | C_7_ H_8_ O_3_ | 17.492 | - | - | 0.19 | - | - | - |
| B43 | 2-Propenal | 107-02-8 | C_3_ H_4_ O | 19.939 | - | - | 0.00 | 0.41 | - | - |
| B44 | Benzoic acid, 2-hydroxy-, propyl ester | 607-90-9 | C_10_ H_12_ O_3_ | 16.17 | - | - | 0.01 | - | - | - |
| B45 | Phenelzine | 51-71-8 | C_8_ H_12_ N_2_ | 3.518 | - | - | 0.01 | - | - | - |
| B46 | Propanoic acid, anhydride | 123-62-6 | C_6_ H_10_ O_3_ | 4.528 | - | - | - | 3.24 | - | - |
| B47 | Pentadecanoic acid, ethyl ester | 41114-00-5 | C_17_ H_34_ O_2_ | 25.944 | - | - | - | 1.05 | - | - |
| B48 | 1-methoxypropan-2-yl 2-cyanoacetate | 32804-79-8 | C7 H11 N O3 | 4.245 | - | - | - | 0.21 | - | 0.19 |
| B49 | Hydroxyurea | 127-07-1 | C H_4_ N_2_ O_2_ | 3.053 | - | - | - | 0.33 | - | - |
| B50 | 2-Propanamine, N,N'-methanetetraylbis- | 693-13-0 | C_7_ H_14_ N_2_ | 23.753 | - | - | - | 0.05 | - | - |
| B51 | 1H-Tetrazole | 288-94-8 | C H_2_ N_4_ | 2.503 | - | - | - | 1.48 | 0.08 | 0.44 |
| B52 | Methyl propyl ether | 557-17-5 | C_4_ H_10_ O | 4.169 | - | - | - | 0.86 | - | - |
| B53 | Octodrine | 105-20-4 | C_5_ H_9_ N_3_ | 25.99 | - | - | - | 0.15 | - | 0.01 |
| B54 | Octodrine | 543-82-8 | C_8_ H_19_ N | 21.078 | - | - | - | 0.15 | - | - |
| B55 | Serine | 56-45-1 | C_3_ H_7_ N O_3_ | 3.813 | - | - | - | 0.02 | - | - |
| B56 | Phenol, 4-[2-(methylamino)ethyl]- | 370-98-9 | C_9_ H_13_ N O | 11.337 | - | - | - | 0.01 | - | - |
| B57 | Sydnone, 3-phenyl- | 120-06-9 | C_8_ H_6_ N_2_ O_2_ | 17.662 | - | - | - | 0.04 | - | - |
| B58 | Hydrastine | 118-08-1 | C_21_ H_21_ N O_6_ | 39.762 | - | - | - | 0.02 | - | - |
| B59 | 9-Decen-1-ol | 13019-22-2 | C_10_ H_20_ O | 20.8 | - | - | - | - | 1.22 | 1.30 |
| B60 | 2-Nonanol | 628-99-9 | C_9_ H_20_ O | 18.315 | - | - | - | - | 1.95 | - |
| B61 | Phenol, Benzyl cinnamate | 103-41-3 | C_16_H_14_O_2_ | 23.166 | - | - | - | - | 0.09 | - |
| B62 | 2-Dodecanone | 6175-49-1 | C_12_ H_24_ O | 22.502 | - | - | - | - | 0.15 | - |
| B63 | Glycolamide | 598-42-5 | C_2_ H_5_ N O_2_ | 2.145 | - | - | - | - | 1.51 | - |
| B64 | 1-Dodecanol | 112-53-8 | C_12_ H_26_ O | 24.281 | - | - | - | - | 0.24 | 0.43 |
| B65 | Benorilate | 5003-48-5 | C_17_ H_15_ N O_5_ | 20.11 | - | - | - | - | 0.01 | - |
| B66 | Naphthalene, 1,2,3,5,6,7,8,8a-octahydro-1,8a-dimethyl-7-(1-methylethenyl)-, [1R-(1.alpha.,7.beta.,8a.alpha.)]- | 4630-07-3 | C_15_ H_24_ | 22.619 | - | - | - | - | 0.10 | - |
| B67 | 2-Pyrrolidinone | 616-45-5 | C_4_ H_7_ N O | 12.739 | - | - | - | - | 0.02 | - |
| B68 | 5-Diazouracil | 2435-76-9 | C_4_ H_2_ N_4_ O_2_ | 19.308 | - | - | - | - | 0.02 | 0.04 |
| B69 | N-Nitrosodimethylamine | 62-75-9 | C_2_ H_6_ N_2_ O | 2.781 | - | - | - | - | 0.02 | - |
| B70 | Butanoic acid, 3-methyl- | 503-74-2 | C_5_ H_10_ O_2_ | 6.167 | - | - | - | - | 0.01 | - |
| B71 | Butyl benzoate | 136-60-7 | C_11_ H_14_ O_2_ | 22.128 | - | - | - | - | - | 0.07 |
| B72 | psi-Baptigenin | 90-29-9 | C_16_ H_10_ O_5_ | 30.761 | - | - | - | - | - | 0.01 |
| B73 | 4H-1-Benzopyran-4-one, 7-hydroxy-2-(4-methoxyphenyl)- | 487-24-1 | C_16_ H_12_ O_4_ | 39.669 | - | - | - | - | - | 0.01 |
| B74 | Irbesartan | 138402-11-6 | C_25_ H_28_ N_6_ O | 37.593 | - | - | - | - | - | 0.01 |
| B75 | Betaine | 107-43-7 | C_5_ H_11_ N O_2_ | 4.736 | - | - | - | - | - | 0.00 |
| B76 | Furfural | 98-01-1 | C_5_ H_4_ O_2_ | 7.15 | - | - | - | - | - | 0.04 |
| B77 | Conessine | 546-06-5 | C_24_ H_40_ N_2_ | 21.794 | - | - | - | - | - | 0.01 |
| B78 | 2-Amino-1,3-propanediol | 534-03-2 | C_3_ H_9_ N O_2_ | 4.579 | - | - | - | - | - | 0.03 |
| B79 | (S)-(+)-Isoleucinol | 24629-25-2 | C_6_ H_15_ N O | 4.644 | - | - | - | - | - | 0.11 |

Table S4 Composition and relative content of ethyl acetate extract of *Salvia plebeia*

| No. | Components | CAS | Molecular formula | Retention Time/min | Relative content/% | |
| --- | --- | --- | --- | --- | --- | --- |
|  |  |  |  |  | Root | Leaf |
| C1 | Propanoic acid, ethyl ester | 105-37-3 | C5 H10 O2 | 2.084 | 25.58 | 21.06 |
| C2 | n-Propyl acetate | 109-60-4 | C5 H10 O2 | 2.1 | 6.65 | 7.95 |
| C3 | Ethane, 1,1-diethoxy- | 105-57-7 | C6 H14 O2 | 2.188 | 3.36 | - |
| C4 | Ethyl orthoformate | 122-51-0 | C7 H16 O3 | 2.835 | 0.13 | 0.10 |
| C5 | Acetic acid, butyl ester | 123-86-4 | C6 H12 O2 | 2.853 | 49.80 | 30.86 |
| C6 | Ethylbenzene | 100-41-4 | C8 H10 | 3.503 | 0.33 | 0.11 |
| C7 | Benzene, 1,3-dimethyl- | 108-38-3 | C8 H10 | 3.61 | 1.42 | 0.82 |
| C8 | Ferruginol | 514-62-5 | C20 H30 O | 52.17 | 5.41 | 13.56 |
| C9 | rac-Ibuprofen Amide | 59512-17-3 | C13 H19 N O | 28.068 | 1.52 | 2.55 |
| C10 | Methane, diethoxy- | 462-95-3 | C5 H12 O2 | 2.841 | 0.13 | - |
| C11 | Pentanoic acid | 109-52-4 | C5 H10 O2 | 5.644 | 0.01 | 0.05 |
| C12 | 8-Azaadenine | 1123-54-2 | C4 H4 N6 | 39.429 | 0.01 | - |
| C13 | n-Hexadecanoic acid | 57-10-3 | C16 H32 O2 | 41.632 | 2.19 | 1.43 |
| C14 | 2,4-Decadienal, (E,E)- | 25152-84-5 | C10 H16 O | 20.332 | 0.10 | - |
| C15 | 2-Hydroxynicotinic acid | 609-71-2 | C6 H5 N O3 | 22.717 | 0.23 | - |
| C16 | 2,4-Imidazolidinedione, 5-phenyl- | 89-24-7 | C9 H8 N2 O2 | 28.881 | 0.08 | - |
| C17 | Isobutyronitrile | 78-82-0 | C4 H7 N | 9.449 | 0.01 | - |
| C18 | Benzophenone | 119-61-9 | C13 H10 O | 29.982 | 0.01 | - |
| C19 | 2-Propanamine, N,N'-methanetetraylbis- | 693-13-0 | C7 H14 N2 | 41.201 | 0.08 | 0.44 |
| C20 | .alpha.-Campholenal | 4501-58-0 | C10 H16 O | 20.321 | 0.66 | - |
| C21 | Propane, 2-isocyano-2-methyl- | 7188-38-7 | C5 H9 N | 32.677 | 0.01 | 0.02 |
| C22 | Pyridine, 4-nitro-, 1-oxide | 1124-33-0 | C5 H4 N2 O3 | 35.553 | 0.01 | - |
| C23 | Ethyl diazoacetate | 623-73-4 | C4 H6 N2 O2 | 35.923 | 0.01 | - |
| C24 | 1,2-Benzenedicarbonitrile | 91-15-6 | C8 H4 N2 | 3.606 | 0.02 | - |
| C25 | Hexanoic acid | 142-62-1 | C6 H12 O2 | 5.65 | 0.02 | - |
| C26 | Isophthalaldehyde | 626-19-7 | C8 H6 O2 | 13.714 | 0.02 | - |
| C27 | Betazole | 105-20-4 | C5 H9 N3 | 16.889 | 0.01 | - |
| C28 | 3-Pyridinecarboxylic acid, 4-hydroxy- | 609-70-1 | C6 H5 N O3 | 22.735 | 0.14 | - |
| C29 | 1H-1,2,3-Triazole | 288-36-8 | C2 H3 N3 | 47.16 | 0.18 | - |
| C30 | Propane, 2-isocyanato- | 1795-48-8 | C4 H7 N O | 2.252 | 0.01 | 0.20 |
| C31 | Ethane, isocyanato- | 109-90-0 | C3 H5 N O | 13.039 | 0.04 | - |
| C32 | 3,5-Di-tert-butyl-2-hydroxybenzaldehyde | 37942-07-7 | C15 H22 O2 | 40.236 | 0.07 | - |
| C33 | 8-Quinolinol, 1-oxide | 1127-45-3 | C9 H7 N O2 | 55.917 | 0.11 | - |
| C34 | Methylal | 109-87-5 | C3 H8 O2 | 2.921 | 0.16 | - |
| C35 | Cyclobutylamine | 2516-34-9 | C4 H9 N | 6.033 | 0.01 | - |
| C36 | 5-Diazouracil | 2435-76-9 | C4 H2 N4 O2 | 22.77 | 0.01 | - |
| C37 | 1,4-Cyclohexanedione | 637-88-7 | C6 H8 O2 | 36.298 | 0.00 | - |
| C38 | 4-Isopropylphenyl isocyanate | 31027-31-3 | C10 H11 N O | 48.937 | 0.16 | - |
| C39 | Benzene, nitroso- | 586-96-9 | C6 H5 N O | 3.513 | 0.09 | - |
| C40 | Propanoic acid, butyl ester | 590-01-2 | C7 H14 O2 | 4.178 | 0.03 | - |
| C41 | Propiolic acid | 471-25-0 | C3 H2 O2 | 36.209 | 0.07 | - |
| C42 | Dicyclopentadiene | 77-73-6 | C10 H12 | 7.046 | 0.11 | - |
| C43 | Cyclohexane, isocyanato- | 3173-53-3 | C7 H11 N O | 47.049 | 1.03 | 1.24 |
| C44 | Cyclooctane | 292-64-8 | C8 H16 | 16.001 | - | 0.09 |
| C45 | 5-Nitroso-2,4,6-triaminopyrimidine | 1006-23-1 | C4 H6 N6 O | 50.707 | - | 0.03 |
| C46 | 1,2,4-Benzotriazin-3-amine, 1,4-dioxide | 27314-97-2 | C7 H6 N4 O2 | 56.158 | - | 0.57 |
| C47 | 2-Octen-4-one | 4643-27-0 | C8 H14 O | 16.006 | - | 0.07 |
| C48 | 1H-Tetrazol-5-amine | 4418-61-5 | C H3 N5 | 22.12 | - | 0.07 |
| C49 | Benzenamine, N,N-diethyl-4-nitroso- | 120-22-9 | C10 H14 N2 O | 49.011 | - | 0.80 |
| C50 | Benzofuroxan | 480-96-6 | C6 H4 N2 O2 | 50.603 | - | 0.12 |
| C51 | 5H-Benzocyclohepten-5-one, 2,3,4,6-tetrahydroxy- | 569-77-7 | C11 H8 O5 | 44.556 | - | 0.26 |
| C52 | Octane | 111-65-9 | C8 H18 | 2.705 | - | 0.19 |
| C53 | 2-Butenoic acid, 3-methyl-, methyl ester | 924-50-5 | C6 H10 O2 | 6.121 | - | 0.03 |
| C54 | Butanoic acid, octyl ester | 110-39-4 | C12 H24 O2 | 23.01 | - | 0.07 |
| C55 | Ethanone, 1-(1H-pyrrol-2-yl)- | 1072-83-9 | C6 H7 N O | 39.382 | - | 0.02 |
| C56 | 2H-1-Benzopyran-2-one, 6-amino- | 14415-44-2 | C9 H7 N O2 | 28.074 | - | 0.32 |
| C57 | Celestolide | 13171-00-1 | C17 H24 O | 48.068 | - | 4.41 |
| C58 | Pimpinellin | 131-12-4 | C13 H10 O5 | 51.695 | - | 0.30 |
| C59 | 2(3H)-Furanone, dihydro-3-methylene- | 547-65-9 | C5 H6 O2 | 18.562 | - | 0.04 |
| C60 | Minoxidil | 38304-91-5 | C9 H15 N5 O | 55.436 | - | 0.02 |
| C61 | 2(5H)-Furanone | 497-23-4 | C4 H4 O2 | 17.624 | - | 0.07 |
| C62 | 2-Pyrrolidinone, 1-phenyl- | 4641-57-0 | C10 H11 N O | 30.518 | - | 0.01 |
| C63 | Neophytadiene | 504-96-1 | C20 H38 | 37.516 | - | 9.21 |
| C64 | Trigonelline | 535-83-1 | C7 H7 N O2 | 43.354 | - | 0.03 |
| C65 | Propane, 2-isocyanato-2-methyl- | 1609-86-5 | C5 H9 N O | 46.193 | - | 0.16 |
| C66 | Benzene, 1-isocyanato-4-nitro- | 100-28-7 | C7 H4 N2 O3 | 48.223 | - | 0.09 |
| C67 | dl-c-Allylglycine | 7685-44-1 | C5 H9 N O2 | 18.574 | - | 0.02 |
| C68 | Pentylenetetrazol | 54-95-5 | C6 H10 N4 | 21.34 | - | 0.01 |
| C69 | Phenol, 4-nitroso- | 104-91-6 | C6 H5 N O2 | 49.578 | - | 0.14 |
| C70 | 2-Propanone, 1,1-dimethoxy- | 6342-56-9 | C5 H10 O3 | 2.921 | - | 0.02 |
| C71 | D-Allothreonine | 24830-94-2 | C4 H9 N O3 | 4.173 | - | 0.02 |
| C72 | N-Acetyltyramine | 1202-66-0 | C10 H13 N O2 | 28.533 | - | 1.85 |
| C73 | 3-Pentanol, 3-methyl- | 77-74-7 | C6 H14 O | 2.594 | - | 0.06 |
| C74 | 1,1'-Carbonyldiimidazole | 530-62-1 | C7 H6 N4 O | 8.371 | - | 0.02 |
| C75 | Pyridine, 2-ethenyl- | 100-69-6 | C7 H7 N | 21.048 | - | 0.01 |
| C76 | .alpha.-Furil | 492-94-4 | C10 H6 O4 | 44.329 | - | 0.21 |
| C77 | Phenethyl isocyanate | 1943-82-4 | C9 H9 N O | 53.424 | - | 0.12 |
| C78 | Ethyl isocyanoacetate | 2999-46-4 | C5 H7 N O2 | 2.264 | - | 0.02 |
| C79 | Xylose | 58-86-6 | C5 H10 O5 | 5.647 | - | 0.04 |
| C80 | (-)-Calamenene | 483-77-2 | C15 H22 | 32.123 | - | 0.10 |

Table S5 Composition and relative content of ethyl acetate extract from fermentation broth of *Salvia plebeia* endophytic bacteria

| No. | Components | CAS | Molecular formula | Retention Time/min | Relative content/% | | | | | |
| --- | --- | --- | --- | --- | --- | --- | --- | --- | --- | --- |
|  |  |  |  |  | L1 | L24 | R86 | R109 | S10 | S26 |
| D1 | Formic acid hydrazide | 624-84-0 | C H_4_ N_2_ O | 3.179 | 3.90 | - | - | - | - | 15.10 |
| D2 | Butane, 2-isocyanato- | 15585-98-5 | C_5_ H_9_ N O | 11.821 | 1.95 | - | 0.45 | - | - | - |
| D3 | 1H-Benzotriazole, 1-methyl- | 13351-73-0 | C_7_ H_7_ N_3_ | 17.009 | 0.21 | - | - | - | - | - |
| D4 | 1-Butanol, 2-methyl-, propanoate | 2438-20-2 | C_8_ H_16_ O_2_ | 21.704 | 0.34 | - | - | - | - | - |
| D5 | 1H-Pyrrole-3-carboxylic acid | 931-03-3 | C_5_ H_5_ N O_2_ | 36.1 | 0.28 | - | - | - | - | - |
| D6 | Cyclobutanecarboxylic acid, 1-amino- | 22264-50-2 | C_5_ H_9_ N O_2_ | 5.63 | 0.14 | 0.04 | - | 0.01 | - | 0.12 |
| D7 | 1,3-Dioxolane | 646-06-0 | C_3_ H_6_ O_2_ | 5.81 | 0.19 | 0.03 | - | - | - | 0.28 |
| D8 | N-1H-Tetrazol-5-ylacetamide | 6158-77-6 | C_3_ H_5_ N_5_ O | 26.935 | 2.21 | - | - | - | - | - |
| D9 | 2,6-Lutidine-N-oxide | 1073-23-0 | C_7_ H_9_ N O | 36.973 | 0.21 | - | - | - | - | - |
| D10 | N,N'-di-tert-Butylcarbodiimide | 691-24-7 | C_9_ H_18_ N_2_ | 48.282 | 0.54 | - | - | - | - | - |
| D11 | 2,5-Dimethoxyphenyl isocyanate | 56309-62-7 | C_9_ H_9_ N O_3_ | 59.413 | 1.09 | - | - | - | - | - |
| D12 | Methyldiethanolamine | 105-59-9 | C_5_ H_13_ N O_2_ | 6.017 | 0.46 | - | - | - | 4.63 | - |
| D13 | 3-Pentanone | 96-22-0 | C_5_ H_10_ O | 7.892 | 0.57 | - | - | - | - | - |
| D14 | 1,4-Dioxane-2,5-dione, 3,6-dimethyl- | 95-96-5 | C_6_ H_8_ O_4_ | 11.958 | 0.09 | - | - | - | - | - |
| D15 | 1H-Benzotriazole | 95-14-7 | C_6_ H_5_ N_3_ | 34.736 | 0.50 | 0.09 | - | - | - | 0.31 |
| D16 | 3-Furaldehyde | 498-60-2 | C_5_ H_4_ O_2_ | 35.491 | 0.31 | 0.03 | - | - | - | - |
| D17 | 1,4-Cyclohexanedione | 637-88-7 | C_6_ H_8_ O_2_ | 36.211 | 1.17 | - | - | - | - | - |
| D18 | (4-Oxo-1,2,3-benzotriazin-3(4H)-yl)acetic acid | 97609-01-3 | C_9_ H_7_ N_3_ O_3_ | 56.443 | 0.45 | - | - | - | - | - |
| D19 | Butanoic acid | 107-92-6 | C_4_ H_8_ O_2_ | 3.178 | 23.48 | - | - | 0.02 | - | - |
| D20 | Pentanoic acid | 109-52-4 | C_5_ H_10_ O_2_ | 4.696 | 0.17 | 0.04 | - | - | - | - |
| D21 | Perhydrofarnesyl Acetone | 502-69-2 | C_18_ H_36_ O | 8.282 | 0.18 | 0.03 | - | - | 44.21 | 0.14 |
| D22 | 5-Diazouracil | 2435-76-9 | C_4_ H_2_ N_4_ O_2_ | 37.195 | 0.32 | - | 0.03 | - | - | - |
| D23 | Pyridine, 1-oxide | 694-59-7 | C_5_ H_5_ N O | 48.168 | 0.17 | 0.05 | - | - | - | - |
| D24 | Aspartic acid | 56-84-8 | C_4_ H_7_ N O_4_ | 3.123 | 2.66 | - | - | - | - | - |
| D25 | Cyclobutanone | 1191-95-3 | C_4_ H_6_ O | 3.19 | 8.12 | - | - | - | - | - |
| D26 | Pyrazine | 290-37-9 | C_4_ H_4_ N_2_ | 4.02 | 0.40 | - | - | - | - | - |
| D27 | Butanoic acid, 3-methyl- | 503-74-2 | C_5_ H_10_ O_2_ | 4.076 | 43.52 | 57.22 | 0.26 | 3.56 | - | 30.07 |
| D28 | 5-Aminopyrimidine | 591-55-9 | C_4_ H_5_ N_3_ | 16.194 | 0.17 | - | - | 0.01 | - | 0.18 |
| D29 | (S)-2,6-Dioxohexahydro-4-pyrimidinecarboxylic acid | 5988-19-2 | C_5_ H_6_ N_2_ O_4_ | 19.933 | 0.68 | - | - | - | - | - |
| D30 | 1,4-Dioxane-2,6-dione | 4480-83-5 | C_4_ H_4_ O_4_ | 22.905 | 0.47 | 0.29 | - | - | - | 6.96 |
| D31 | Propane, 1-isocyanato- | 110-78-1 | C_4_ H_7_ N O | 24.79 | 0.76 | - | - | 0.63 | - | 1.71 |
| D32 | Phenol, 2-amino-4,6-bis(1,1-dimethylethyl)- | 1643-39-6 | C_14_ H_23_ N O | 28.999 | 3.17 | - | - | - | - | - |
| D33 | Pirenzepine | 28797-61-7 | C_19_ H_21_ N_5_ O_2_ | 38.046 | 0.75 | - | - | - | - | - |
| D34 | 3-Pyridinol, 4-methyl-, acetate (ester) | 1006-96-8 | C_8_ H_9_ N O_2_ | 48.352 | 0.10 | - | - | - | - | - |
| D35 | Benzene, ethoxy- | 103-73-1 | C_8_ H_10_ O | 50.691 | 0.28 | - | 0.03 | - | 0.82 | 0.08 |
| D36 | 2-Butynoic acid | 590-93-2 | C_4_ H_4_ O_2_ | 16.381 | - | 0.04 | 0.05 | - | 0.40 | - |
| D37 | Methyl pyrrole-2-carboxylate | 1193-62-0 | C_6_ H_7_ N O_2_ | 33.664 | - | 0.05 | - | - | 0.71 | - |
| D38 | Pentylenetetrazol | 54-95-5 | C_6_ H_10_ N_4_ | 34.305 | - | 0.09 | 0.07 | - | 0.60 | - |
| D39 | 2-Amino-1,3-propanediol | 534-03-2 | C_3_ H_9_ N O_2_ | 4.459 | - | 4.31 | - | - | - | - |
| D40 | 3-Amino-4-methyl-pentanoic acid | 5699-54-7 | C_6_ H_13_ N O_2_ | 4.479 | - | 0.76 | - | - | - | - |
| D41 | Xylose | 58-86-6 | C_5_ H_10_ O_5_ | 6.632 | - | 0.05 | - | - | - | - |
| D42 | .beta.-D-Glucopyranose, 1,6-anhydro- | 498-07-7 | C_6_ H_10_ O_5_ | 8.777 | - | 0.04 | - | - | - | - |
| D43 | Benzeneacetaldehyde | 122-78-1 | C_8_ H_8_ O | 9.662 | - | 0.50 | - | - | - | - |
| D44 | Acetic acid, methyl ester | 79-20-9 | C_3_ H_6_ O_2_ | 11.893 | - | 0.02 | - | - | - | 0.07 |
| D45 | Formamide, N,N-dimethyl- | 68-12-2 | C_3_ H_7_ N O | 12.239 | - | 0.04 | - | - | - | 0.07 |
| D46 | 4-Isopropoxybenzoic acid | 13205-46-4 | C_10_ H_12_ O_3_ | 27.126 | - | 0.35 | - | - | - | - |
| D47 | 8-Azahypoxanthine | 2683-90-1 | C_4_ H_3_ N_5_ O | 37.814 | - | 0.09 | - | 0.02 | - | - |
| D48 | Pyrrolo[1,2-a]pyrazine-1,4-dione, hexahydro-3-(2-methylpropyl)- | 5654-86-4 | C_11_ H_18_ N_2_ O_2_ | 43.704 | - | 0.95 | 7.70 | 1.22 | - | - |
| D49 | Benzene, 1-isocyanato-4-nitro- | 100-28-7 | C_7_ H_4_ N_2_ O_3_ | 57.336 | - | 0.26 | - | - | - | - |
| D50 | Iminodiacetic acid | 142-73-4 | C_4_ H_7_ N O_4_ | 3.475 | - | 0.14 | 1.63 | 1.18 | - | - |
| D51 | Methanamine, N,N-dimethyl-, N-oxide | 1184-78-7 | C_3_ H_9_ N O | 4.519 | - | 8.83 | - | - | - | - |
| D52 | Acetic acid, methoxy-, methyl ester | 6290-49-9 | C_4_ H_8_ O_3_ | 5.406 | - | 0.05 | - | - | - | - |
| D53 | (S)-(+)-2-Amino-3-methyl-1-butanol | 2026-48-4 | C_5_ H_13_ N O | 10.327 | - | 0.03 | - | - | - | - |
| D54 | Propanamide | 79-05-0 | C_3_ H_7_ N O | 11.955 | - | 0.05 | - | - | - | - |
| D55 | L-Alanine, N-benzoyl-, methyl ester | 7244-67-9 | C_11_ H_13_ N O_3_ | 12.127 | - | 0.18 | - | - | - | - |
| D56 | Phenethyl isocyanate | 1943-82-4 | C_9_ H_9_ N O | 12.145 | - | 0.81 | 0.14 | 0.08 | 2.42 | - |
| D57 | Isoxazole | 288-14-2 | C_3_ H_3_ N O | 13.346 | - | 0.04 | - | - | - | - |
| D58 | 1-Propanol, 3-(phenylmethoxy)- | 4799-68-2 | C_10_ H_14_ O_2_ | 14.296 | - | 0.05 | - | - | - | - |
| D59 | Guanidine | 113-00-8 | C H_5_ N_3_ | 14.32 | - | 0.13 | - | 0.60 | - | - |
| D60 | dl-c-Allylglycine | 7685-44-1 | C_5_ H_9_ N O_2_ | 16.511 | - | 0.09 | - | - | - | 1.13 |
| D61 | Butanoic acid, 1,1-dimethyl-2-phenylethyl ester | 10094-34-5 | C_14_ H_20_ O_2_ | 16.523 | - | 0.09 | - | - | 3.83 | - |
| D62 | 1H-Pyrazole | 288-13-1 | C_3_ H_4_ N_2_ | 31.828 | - | 0.02 | 0.01 | - | - | - |
| D63 | Pyrrolo[1,2-a]pyrazine-1,4-dione, hexahydro- | 19179-12-5 | C_7_ H_10_ N_2_ O_2_ | 37.852 | - | 15.91 | 78.38 | 82.53 | - | 31.03 |
| D64 | 4-Nitro-3-picoline-N-oxide | 1074-98-2 | C_6_ H_6_ N_2_ O_3_ | 37.86 | - | 7.40 | - | - | - | - |
| D65 | 2-Pyridinamine, 4-methyl- | 695-34-1 | C_6_ H_8_ N_2_ | 43.993 | - | 0.03 | - | - | - | - |
| D66 | Serine | 56-45-1 | C_3_ H_7_ N O_3_ | 9.39 | - | 0.03 | - | - | - | - |
| D67 | 2,6-Pyridinedicarboxylic acid | 499-83-2 | C_7_ H_5_ N O_4_ | 26.839 | - | 0.04 | - | - | - | - |
| D68 | 2(5H)-Furanone | 497-23-4 | C_4_ H_4_ O_2_ | 29.901 | - | 0.02 | - | - | - | - |
| D69 | 1,1'-Carbonyldiimidazole | 530-62-1 | C_7_ H_6_ N_4_ O | 31.699 | - | 0.04 | - | 0.01 | - | - |
| D70 | Benzophenone | 119-61-9 | C_13_ H_10_ O | 33.299 | - | 0.11 | - | - | 0.90 | - |
| D71 | Pyrazinamide | 98-96-4 | C_5_ H_5_ N_3_ O | 39.636 | - | 0.03 | - | - | - | - |
| D72 | Cyclohexane, isocyanato- | 3173-53-3 | C_7_ H_11_ N O | 46.031 | - | 0.19 | - | - | - | - |
| D73 | Pyrene | 129-00-0 | C_16_ H_10_ | 48.993 | - | 0.33 | - | - | - | - |
| D74 | 3-Pyridinecarboxylic acid, 4-hydroxy- | 609-70-1 | C_6_ H_5_ N O_3_ | 23.655 | - | - | 0.03 | - | 0.21 | - |
| D75 | Benzene, nitroso- | 586-96-9 | C_6_ H_5_ N O | 25.77 | - | - | 0.03 | - | - | - |
| D76 | 8-Azaxanthine | 1468-26-4 | C_4_ H_3_ N_5_ O_2_ | 44.58 | - | - | 0.21 | 0.06 | 2.01 | - |
| D77 | 8-Quinolinol, 1-oxide | 1127-45-3 | C_9_ H_7_ N O_2_ | 44.72 | - | - | 0.10 | 0.02 | - | - |
| D78 | 2-Propanamine, 2-methyl-N-(phenylmethylene)-, N-oxide | 3376-24-7 | C_11_ H_15_ N O | 49.963 | - | - | 0.11 | - | 1.00 | - |
| D79 | Benzene, 2-isocyanato-1,3-bis(1-methylethyl)- | 28178-42-9 | C_13_ H_17_ N O | 51.221 | - | - | 0.08 | - | - | - |
| D80 | Hexanoic acid, 2-methyl- | 4536-23-6 | C_7_ H_14_ O_2_ | 4.193 | - | - | 0.83 | - | - | - |
| D81 | 2-Amino-5-methylbenzoic acid | 2941-78-8 | C_8_ H_9_ N O_2_ | 5.266 | - | - | 0.11 | 0.22 | - | - |
| D82 | 3-Hexen-1-ol, propanoate, (Z)- | 33467-74-2 | C_9_ H_16_ O_2_ | 18.282 | - | - | 0.03 | - | - | - |
| D83 | 1H-Pyrazole, 4-nitro- | 2075-46-9 | C_3_ H_3_ N_3_ O_2_ | 25.16 | - | - | 0.28 | - | - | - |
| D84 | Butylated Hydroxytoluene | 128-37-0 | C_15_ H_24_ O | 28.985 | - | - | 0.61 | 0.50 | - | - |
| D85 | 2,6-Pyridinediamine | 141-86-6 | C_5_ H_7_ N_3_ | 30.206 | - | - | 0.04 | - | - | - |
| D86 | Myristic acid vinyl ester | 5809-91-6 | C_16_ H_30_ O_2_ | 34.956 | - | - | 0.26 | - | - | - |
| D87 | 8-(2-Phenylethyl)-1-oxa-3,8-diazaspiro[4.5]decan-2-one | 5053-06-5 | C_15_ H_20_ N_2_ O_2_ | 39.493 | - | - | 0.57 | - | - | - |
| D88 | Cyclo(L-prolyl-L-valine) | 2854-40-2 | C_10_ H_16_ N_2_ O_2_ | 39.941 | - | - | 0.87 | - | - | - |
| D89 | 1-Propanol, 3-(diethylamino)-2,2-dimethyl-, p-aminobenzoate (ester) | 94-15-5 | C_16_ H_26_ N_2_ O_2_ | 43.7 | - | - | 0.25 | 0.03 | - | - |
| D90 | 1H-Isoindole-1,3(2H)-dione, 2-phenyl- | 520-03-6 | C_14_ H_9_ N O_2_ | 57.018 | - | - | 1.71 | - | - | - |
| D91 | Aminopyrazine | 5049-61-6 | C_4_ H_5_ N_3_ | 5.468 | - | - | 0.04 | 0.02 | 0.51 | - |
| D92 | O-Methylisourea | 2440-60-0 | C_2_ H_6_ N_2_ O | 8.314 | - | - | 0.02 | - | - | - |
| D93 | Benzofuroxan | 480-96-6 | C_6_ H_4_ N_2_ O_2_ | 18.338 | - | - | 0.23 | - | - | - |
| D94 | Benzaldehyde, 2,4-dihydroxy- | 95-01-2 | C_7_ H_6_ O_3_ | 21.993 | - | - | 0.04 | - | - | - |
| D95 | 2-Octanol, 8,8-dimethoxy-2,6-dimethyl- | 141-92-4 | C_12_ H_26_ O_3_ | 22.12 | - | - | 0.04 | - | - | - |
| D96 | Sarcosine ethyl ester hydrochloride | 52605-49-9 | C_5_ H_11_ N O_2_ | 24.354 | - | - | 0.01 | - | - | - |
| D97 | 3-Amino-s-triazole | 61-82-5 | C_2_ H_4_ N_4_ | 31.317 | - | - | 0.05 | - | - | - |
| D98 | Glycyl-L-tyrosine | 658-79-7 | C_11_ H_14_ N_2_ O_4_ | 31.329 | - | - | 0.02 | - | - | - |
| D99 | 6-Aminonicotinamide | 329-89-5 | C_6_ H_7_ N_3_ O | 38.098 | - | - | 0.04 | - | - | - |
| D100 | Benzeneacetic acid, ethyl ester | 101-97-3 | C_10_ H_12_ O_2_ | 40.034 | - | - | 0.17 | - | - | - |
| D101 | Minoxidil | 38304-91-5 | C_9_ H_15_ N_5_ O | 54.11 | - | - | 0.27 | - | - | 0.46 |
| D102 | N-.omega.-Acetylhistamine | 673-49-4 | C_7_ H_11_ N_3_ O | 8.496 | - | - | 0.01 | 0.01 | - | 0.26 |
| D103 | Butanedioic acid, 2,3-dihydroxy- [R-(R*,R*)]-, dimethyl ester | 608-68-4 | C_6_ H_10_ O_6_ | 12.432 | - | - | 0.03 | - | - | - |
| D104 | 3-Aminoacetophenone | 99-03-6 | C_8_ H_9_ N O | 20.231 | - | - | 1.30 | - | - | 0.19 |
| D105 | Barbituric acid | 67-52-7 | C_4_ H_4_ N_2_ O_3_ | 25.116 | - | - | 0.33 | - | - | - |
| D106 | Formamide, N-methyl-N-phenyl- | 93-61-8 | C_8_ H_9_ N O | 31.121 | - | - | 0.05 | 0.01 | - | - |
| D107 | Benzenamine, 4-(4-morpholinyl)- | 2524-67-6 | C_10_ H_14_ N_2_ O | 36.282 | - | - | 0.08 | - | - | - |
| D108 | 2-Butanone, 4-(4-hydroxy-3-methoxyphenyl)- | 122-48-5 | C_11_ H_14_ O_3_ | 36.402 | - | - | 0.10 | - | - | 0.38 |
| D109 | Benzoic acid, 3-methyl-, methyl ester | 99-36-5 | C_9_ H_10_ O_2_ | 38.111 | - | - | 0.04 | - | - | - |
| D110 | Ethyl diazoacetate | 623-73-4 | C_4_ H_6_ N_2_ O_2_ | 44.964 | - | - | 0.74 | 0.02 | 1.23 | - |
| D111 | Scoparone | 120-08-1 | C_11_ H_10_ O_4_ | 47.307 | - | - | 0.15 | - | - | - |
| D112 | Furfurylamine, .alpha.-benzyl-N-ethyl-tetrahydro-, D-threo- | 3563-92-6 | C_14_ H_21_ N O | 47.557 | - | - | 0.03 | - | - | - |
| D113 | Pyrrolo[1,2-a]pyrazine-1,4-dione, hexahydro-3-(phenylmethyl)- | 14705-60-3 | C_14_ H_16_ N_2_ O_2_ | 56.691 | - | - | 1.34 | - | - | - |
| D114 | Benzenemethanol, .alpha.-methyl- | 98-85-1 | C_8_ H_10_ O | 12.381 | - | - | - | 1.60 | - | - |
| D115 | ortho-Hydroxypropiophenone | 610-99-1 | C_9_ H_10_ O_2_ | 22.508 | - | - | - | 0.05 | - | - |
| D116 | Formetorex | 22148-75-0 | C_10_ H_13_ N O | 11.921 | - | - | - | 0.03 | - | - |
| D117 | 1H-Pyrrole-2,5-dione, 1,1'-(1,2-ethanediyl)bis- | 5132-30-9 | C_10_ H_8_ N_2_ O_4_ | 24.067 | - | - | - | 0.03 | - | - |
| D118 | Phenol, 4-ethyl- | 123-07-9 | C_8_ H_10_ O | 25.586 | - | - | - | 0.39 | - | - |
| D119 | 3,4-Dihydroxy-5-methoxybenzaldehyde | 3934-87-0 | C_8_ H_8_ O_4_ | 27.701 | - | - | - | 0.01 | - | - |
| D120 | Phenol, 3,5-bis(1,1-dimethylethyl)- | 1138-52-9 | C_14_ H_22_ O | 28.999 | - | - | - | 1.61 | - | - |
| D121 | Benzyldiethyl-(2,6-xylylcarbamoylmethyl)-ammonium benzoate | 3734-33-6 | C_28_ H_34_ N_2_ O_3_ | 29.176 | - | - | - | 0.24 | - | - |
| D122 | 3,4-Hexanedione | 4437-51-8 | C_6_ H_10_ O_2_ | 35.715 | - | - | - | 0.08 | - | - |
| D123 | Quinoline, 4-nitro-, 1-oxide | 56-57-5 | C_9_ H_6_ N_2_ O_3_ | 54.309 | - | - | - | 0.07 | - | - |
| D124 | Mexiletine | 31828-71-4 | C_11_ H_17_ N O | 7.684 | - | - | - | 0.01 | - | - |
| D125 | Butanoic acid, anhydride | 106-31-0 | C_8_ H_14_ O_3_ | 18.633 | - | - | - | 0.16 | - | - |
| D126 | Uracil | 66-22-8 | C_4_ H_4_ N_2_ O_2_ | 32.722 | - | - | - | 0.05 | - | - |
| D127 | Molsidomine | 25717-80-0 | C_9_ H_14_ N_4_ O_4_ | 50.968 | - | - | - | 1.46 | - | - |
| D128 | Trigonelline | 535-83-1 | C_7_ H_7_ N O_2_ | 52.041 | - | - | - | 0.08 | - | - |
| D129 | 10(9H)-Acridineacetic acid, 9-oxo- | 38609-97-1 | C_15_ H_11_ N O_3_ | 55.271 | - | - | - | 0.03 | 1.02 | - |
| D130 | 2,3-Butanedione | 431-03-8 | C_4_ H_6_ O_2_ | 4.545 | - | - | - | 0.06 | - | - |
| D131 | Ethylmalonic acid | 601-75-2 | C_5_ H_8_ O_4_ | 4.621 | - | - | - | 0.01 | - | - |
| D132 | Tetracyanoethylene | 670-54-2 | C_6_ N_4_ | 15.117 | - | - | - | 0.04 | - | - |
| D133 | 1H-Imidazol-1-ylacetic acid | 22884-10-2 | C_5_ H_6_ N_2_ O_2_ | 21.288 | - | - | - | 0.04 | - | 0.13 |
| D134 | Tromethamine | 77-86-1 | C_4_ H_11_ N O_3_ | 21.638 | - | - | - | 0.01 | - | - |
| D135 | 6-Amino-1,3-dimethyluracil | 6642-31-5 | C_6_ H_9_ N_3_ O_2_ | 27.443 | - | - | - | 0.06 | - | - |
| D136 | Maltol | 118-71-8 | C_6_ H_6_ O_3_ | 34.158 | - | - | - | 0.16 | 1.30 | 0.17 |
| D137 | Benzeneacetic acid, 3-hydroxy- | 621-37-4 | C_8_ H_8_ O_3_ | 36.714 | - | - | - | 0.01 | - | - |
| D138 | Phenol, 2-ethoxy- | 94-71-3 | C_8_ H_10_ O_2_ | 38.099 | - | - | - | 0.08 | - | - |
| D139 | 1H-Pyrrole-2-acetic acid, 1-methyl-, methyl ester | 51856-79-2 | C_8_ H_11_ N O_2_ | 39.412 | - | - | - | 0.03 | - | - |
| D140 | 1H-Indole, 7-methoxy- | 3189-22-8 | C_9_ H_9_ N O | 42.378 | - | - | - | 0.04 | - | - |
| D141 | Squalane | 111-01-3 | C_30_ H_62_ | 43.637 | - | - | - | 2.78 | - | - |
| D142 | Benzeneacetic acid, 2-propenyl ester | 1797-74-6 | C_11_ H_12_ O_2_ | 6.003 | - | - | - | - | 0.25 | - |
| D143 | 2-Butenoic acid, ethyl ester, (E)- | 623-70-1 | C_6_ H_10_ O_2_ | 20.258 | - | - | - | - | 0.64 | - |
| D144 | 4(1H)-Pyridone | 108-96-3 | C_5_ H_5_ N O | 37.393 | - | - | - | - | 0.32 | - |
| D145 | Resorcinol, 2-acetyl- | 699-83-2 | C_8_ H_8_ O_3_ | 41.538 | - | - | - | - | 1.31 | - |
| D146 | Benzene, 1-methyl-3-nitro- | 99-08-1 | C_7_ H_7_ N O_2_ | 42.828 | - | - | - | - | 1.25 | - |
| D147 | Butyric acid, 2-hydroxy-3-methyl-, methyl ester | 17417-00-4 | C_6_ H_12_ O_3_ | 4.31 | - | - | - | - | 0.51 | - |
| D148 | Cyclobutylamine | 2516-34-9 | C_4_ H_9_ N | 17.942 | - | - | - | - | 0.30 | - |
| D149 | Thymine | 65-71-4 | C_5_ H_6_ N_2_ O_2_ | 19.368 | - | - | - | - | 1.90 | - |
| D150 | Propiolic acid | 471-25-0 | C_3_ H_2_ O_2_ | 34.772 | - | - | - | - | 1.01 | - |
| D151 | Phenazine, 5-oxide | 304-81-4 | C_12_ H_8_ N_2_ O | 36.315 | - | - | - | - | 4.43 | - |
| D152 | 2-Furancarboxylic acid | 88-14-2 | C_5_ H_4_ O_3_ | 40.998 | - | - | - | - | 1.70 | - |
| D153 | S-(-)-1,1-Diphenylprolinol | 112068-01-6 | C_17_ H_19_ N O | 3.107 | - | - | - | - | 12.54 | - |
| D154 | Pyridine, 2-ethenyl- | 100-69-6 | C_7_ H_7_ N | 16.581 | - | - | - | - | 2.29 | - |
| D155 | Dibenzofuran | 132-64-9 | C_12_ H_8_ O | 28.799 | - | - | - | - | 1.07 | - |
| D156 | 2-Pyridinamine, 6-methyl- | 1824-81-3 | C_6_ H_8_ N_2_ | 32.059 | - | - | - | - | 0.22 | - |
| D157 | Pyrazine, ethyl- | 13925-00-3 | C_6_ H_8_ N_2_ | 32.238 | - | - | - | - | 0.31 | 0.16 |
| D158 | 2-(Diethylamino)acetonitrile | 3010-02-4 | C_6_ H_12_ N_2_ | 39.034 | - | - | - | - | 0.31 | - |
| D159 | (2R)-2-{[(Benzyloxy)carbonyl]amino}-4-tert-butoxy-4-oxobutanoic acid | 71449-08-6 | C_16_ H_21_ N O_6_ | 40.459 | - | - | - | - | 1.99 | - |
| D160 | 2,6-Dihydroxy-7-methylpurine | 552-62-5 | C_6_ H_6_ N_4_ O_2_ | 41.788 | - | - | - | - | 0.23 | - |
| D161 | Mephenytoin | 50-12-4 | C_12_ H_14_ N_2_ O_2_ | 43.295 | - | - | - | - | 0.77 | - |
| D162 | Benzeneacetic acid, 4-hydroxy-, methyl ester | 14199-15-6 | C_9_ H_10_ O_3_ | 49.837 | - | - | - | - | 0.85 | - |
| D163 | Acetic acid, diethoxy-, ethyl ester | 6065-82-3 | C_8_ H_16_ O_4_ | 15.783 | - | - | - | - | - | 0.11 |
| D164 | 2(3H)-Furanone, dihydro-5-propyl- | 105-21-5 | C_7_ H_12_ O_2_ | 18.043 | - | - | - | - | - | 0.69 |
| D165 | p-Benzoquinone, 2-methyl- | 553-97-9 | C_7_ H_6_ O_2_ | 41.318 | - | - | - | - | - | 0.10 |
| D166 | Ethyl isocyanoacetate | 2999-46-4 | C_5_ H_7_ N O_2_ | 5.801 | - | - | - | - | - | 0.15 |
| D167 | Benzamide | 55-21-0 | C_7_ H_7_ N O | 37.089 | - | - | - | - | - | 0.18 |
| D168 | Benzaldehyde, 2-hydroxy-4-methoxy- | 673-22-3 | C_8_ H_8_ O_3_ | 37.888 | - | - | - | - | - | 0.66 |
| D169 | Enfenamic acid | 23049-93-6 | C_15_ H_15_ N O_2_ | 43.369 | - | - | - | - | - | 0.08 |
| D170 | Ethanedioic acid, dimethyl ester | 553-90-2 | C_4_ H_6_ O_4_ | 3.764 | - | - | - | - | - | 0.23 |
| D171 | 1H-Tetrazole-1-acetic acid | 21732-17-2 | C_3_ H_4_ N_4_ O_2_ | 4.334 | - | - | - | - | - | 6.13 |
| D172 | Cyclopropylacetylene | 6746-94-7 | C_5_ H_6_ | 8.959 | - | - | - | - | - | 0.38 |
| D173 | 4,5-Diamino-6-hydroxypyrimidine | 1672-50-0 | C_4_ H_6_ N_4_ O | 18.03 | - | - | - | - | - | 0.34 |
| D174 | 4-Nitro-2-picoline N-oxide | 5470-66-6 | C_6_ H_6_ N_2_ O_3_ | 23.365 | - | - | - | - | - | 0.34 |
| D175 | Phenol, 3,4,5-trimethoxy- | 642-71-7 | C_9_ H_12_ O_4_ | 24.386 | - | - | - | - | - | 0.60 |
| D176 | 2-Furanmethanol, tetrahydro-, acetate | 637-64-9 | C_7_ H_12_ O_3_ | 29.721 | - | - | - | - | - | 0.55 |
| D177 | Benzoic acid, 3,5-dihydroxy- | 99-10-5 | C_7_ H_6_ O_4_ | 43.175 | - | - | - | - | - | 0.39 |
| D178 | 4-Pyridinecarboxamide | 1453-82-3 | C_6_ H_6_ N_2_ O | 49.766 | - | - | - | - | - | 0.07 |

**2 Supplementary Figures**

**2.1 Physicochemical properties of endophytes in *S. plebeia***


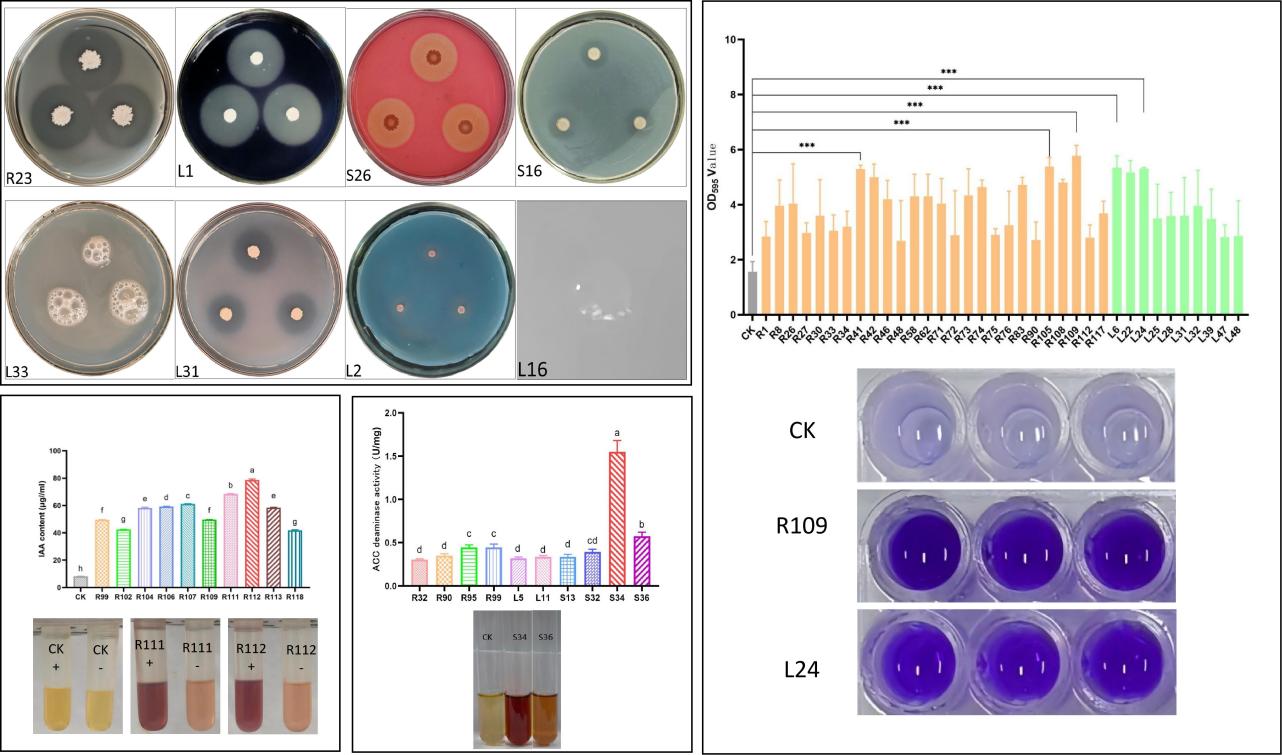


A

H

G

F

E

D

C

B

I

G

K

**Supplementary Figure 1.** Functional characteristics of endophytes from Salvia plebeia. (A: Endophyte with the strongest protease activity; B: Endophyte with the maximum amylase activity; C: Endophyte with the strongest cellulase activity; D: Endophyte with the strongest chitinase activity; E: Endophyte with the maximum catalase activity; F: Endophyte with the strongest phosphate-solubilizing capacity; G: Endophyte with the strongest siderophore-producing capacity; H: Endophyte with the maximum nitrogen-fixing capacity; I: High IAA-producing endophytes; J: Endophytes with relatively strong ACC deaminase activity; K: Endophytes with relatively strong biofilm adhesion.)
